# The Silent Face of Atrial Fibrillation: A Systematic Review of Associated Factors, Clinical Phenotype, and Outcomes

**DOI:** 10.3390/jcm15208191

**Published:** 2026-10-21

**Authors:** Andrea Sonaglioni, Giulio Francesco Gramaglia, Elena Mondazzi, Gian Luigi Nicolosi, Michele Lombardo, Massimo Baravelli

**Affiliations:** 1 Division of Cardiology, IRCCS MultiMedica, 20123 Milan, Italy; michele.lombardo@multimedica.it (M.L.); massimo.baravelli@multimedica.it (M.B.); 2 Department of Emergency, Fondazione IRCSS Ca’ Granda, Ospedale Maggiore Policlinico, 20122 Milan, Italy; giulio.gramaglia@unimi.it (G.F.G.); elena.mondazzi@unimi.it (E.M.); 3 Division of Cardiology, Policlinico San Giorgio, 33170 Pordenone, Italy; gianluigi.nicolosi@gmail.com

**Keywords:** atrial fibrillation, asymptomatic atrial fibrillation, silent atrial fibrillation, subclinical atrial fibrillation, symptom status, atrial remodeling, cardiovascular outcomes, heart failure, systematic review

## Abstract

**Background:** Asymptomatic atrial fibrillation (AF) is increasingly recognized through incidental diagnosis and continuous rhythm monitoring, yet its clinical phenotype and prognostic significance remain incompletely characterized. We systematically evaluated factors associated with asymptomatic AF and its clinical outcomes. **Methods:** This systematic review was conducted according to PRISMA 2020 recommendations. PubMed/MEDLINE, Scopus, and EMBASE were searched from inception through 1 September 2026. Studies reporting demographic, clinical, anthropometric, laboratory, electrocardiographic, imaging, or therapeutic correlates of asymptomatic AF and/or associated clinical outcomes were eligible. Given substantial clinical and methodological heterogeneity, evidence was synthesized using structured qualitative analyses and descriptive summaries of study-level data, without formal meta-analysis or inferential testing of aggregated between-group differences. **Results:** Twenty-three studies were included, comprising 47,456 patients with asymptomatic or subclinical AF and 126,894 participants in the corresponding comparator groups. Study-specific evidence identified male sex, lower ventricular rate, and long-standing persistent or permanent AF as relevant correlates of asymptomatic presentation. Greater left atrial remodeling, including larger atrial dimensions and impaired mechanical function, was also reported in selected studies of silent AF, whereas conventional left ventricular structure and systolic function showed relatively similar descriptive estimates across the available datasets. Selected studies identified metabolic biomarkers and thoracic morphology as additional potential correlates. Rhythm-control strategies and catheter ablation showed numerically lower utilization in descriptive summaries of asymptomatic AF, although the contributing populations were heterogeneous and not necessarily directly comparable. Prognostic findings were heterogeneous: mortality and thromboembolic outcomes were generally comparable between asymptomatic and symptomatic AF in several individual studies, whereas other investigations reported a greater burden of heart failure and heart failure-related events among symptomatic patients. **Conclusions:** Asymptomatic AF represents a heterogeneous clinical phenotype rather than a benign form of the arrhythmia. A lower ventricular response may attenuate symptom perception, while atrial remodeling and cardiovascular risk may progress despite the absence of symptoms. Symptom status should therefore not replace established cardiovascular risk assessment. Thoracic geometry may represent an additional determinant of symptom perception, although current evidence remains preliminary and requires prospective validation.

## 1. Introduction

Atrial fibrillation (AF) is the most common sustained cardiac arrhythmia encountered in clinical practice and represents a major contributor to stroke, heart failure (HF), hospitalization, impaired quality of life, and mortality [1,2,3]. Its clinical presentation is remarkably heterogeneous, ranging from highly symptomatic episodes characterized by palpitations, dyspnea, fatigue, exercise intolerance, or chest discomfort to completely silent forms detected incidentally during routine examination, prolonged electrocardiographic monitoring, or interrogation of implanted cardiac devices [4,5,6]. The increasing availability of wearable technologies, continuous rhythm monitoring, and cardiac implantable electronic devices has further expanded recognition of AF occurring in the absence of overt symptoms, making asymptomatic and subclinical AF an increasingly relevant component of contemporary cardiovascular practice [7,8,9].

The absence of symptoms poses a particular clinical challenge. Symptoms frequently trigger medical evaluation and therefore facilitate AF recognition, whereas asymptomatic AF may remain undiagnosed for prolonged periods and occasionally become apparent only after the occurrence of cardiovascular complications [10,11,12]. Moreover, perception of AF is influenced by multiple factors and does not necessarily parallel arrhythmia burden or underlying cardiovascular disease. Age, sex, ventricular rate, AF pattern and duration, associated cardiovascular conditions, autonomic response, functional status, and individual perception of rhythm disturbances may all contribute to whether AF becomes clinically apparent [13,14,15,16,17,18]. Structural and functional cardiac characteristics, particularly left atrial remodeling [19,20,21], have also emerged as potentially relevant correlates, while more recent evidence has extended investigation to circulating biomarkers [22], anthropometric features [23], and thoracic morphology [24].

This heterogeneity is mirrored by uncertainty regarding the prognostic significance of an asymptomatic presentation. Historically, absence of AF-related symptoms could be perceived as indicating a less clinically relevant arrhythmia. However, symptoms themselves are not included among the principal determinants used to estimate thromboembolic risk, and an asymptomatic presentation does not necessarily imply a low cardiovascular-risk profile. Previous observational studies and registries have produced discordant findings: some have associated asymptomatic AF with increased mortality, stroke, or subsequent AF progression, whereas others have reported risks comparable with, or occasionally lower than, those observed in symptomatic AF [25,26,27,28]. Differences in study setting, patient selection, AF detection methods, symptom classification, background comorbidities, treatment patterns, comparator groups, and duration of follow-up may contribute substantially to these apparently conflicting observations.

A further issue is the close relationship between symptom status and therapeutic management. Decisions regarding rhythm control have traditionally been strongly influenced by symptom burden, and patients without AF-related symptoms may consequently undergo rhythm-control interventions less frequently [29,30]. At the same time, anticoagulation and other strategies aimed at preventing AF-related complications are primarily determined by cardiovascular and thromboembolic risk rather than symptom perception [31,32]. Distinguishing characteristics associated with the absence of symptoms from those reflecting the underlying severity and prognosis of AF is therefore clinically important, particularly as increasingly sensitive monitoring strategies identify a growing population of individuals with previously unrecognized arrhythmia.

Despite the expanding literature, the phenotype of asymptomatic AF remains incompletely characterized. Available evidence encompasses markedly different populations, including community cohorts, hospitalized patients, established AF registries, individuals with persistent or paroxysmal AF, and patients undergoing continuous or device-based rhythm monitoring. Furthermore, demographic and clinical characteristics, biomarkers, cardiac structure and function, AF management, and clinical outcomes have often been examined separately, limiting an integrated assessment of the factors accompanying an asymptomatic presentation and their potential prognostic implications.

Accordingly, the present systematic review was undertaken with two complementary aims: to comprehensively identify demographic, clinical, anthropometric, laboratory, electrocardiographic, imaging, and therapeutic factors associated with asymptomatic AF, and to systematically evaluate the clinical outcomes reported in patients with asymptomatic AF compared with symptomatic AF or other relevant study-specific comparator groups. By integrating evidence across different AF populations and detection settings, this review seeks to provide a comprehensive characterization of the asymptomatic AF phenotype and to clarify the clinical significance of the absence of AF-related symptoms.

## 2. Materials and Methods

### 2.1. Study Design, Reporting Framework, and Protocol Registration

This systematic review was designed, conducted, and reported in accordance with the Preferred Reporting Items for Systematic Reviews and Meta-Analyses (PRISMA) 2020 recommendations [33] (Supplementary Material File S1). The review protocol was registered in the International Platform of Registered Systematic Review and Meta-analysis Protocols (INPLASY) on 3 September 2026 (INPLASY202690012) (Supplementary Material File S2). The protocol registration was non-prospective, as it occurred after completion of the literature search.

### 2.2. Literature Search Strategy

A comprehensive literature search was performed in PubMed, Scopus, and EMBASE from database inception through 1 September 2026. The search strategy was designed to identify studies investigating factors associated with asymptomatic AF as well as its clinical and prognostic outcomes. Search terms included combinations of “atrial fibrillation”, “asymptomatic atrial fibrillation”, “silent atrial fibrillation”, “subclinical atrial fibrillation”, “symptomatic atrial fibrillation”, “asymptomatic status”, “symptom status”, “AF symptoms”, “factors associated with asymptomatic atrial fibrillation”, “predictors”, “determinants”, “risk factors”, “clinical characteristics”, “atrial remodeling”, “outcomes”, “prognosis”, “mortality”, “stroke”, “systemic embolism”, “heart failure”, “hospitalization”, “bleeding”, and “atrial fibrillation progression”. Boolean operators AND and OR were applied as appropriate, and the search syntax was adapted to the indexing structure and requirements of each database. The database-specific search strategies, reconstructed from the search concepts used in the review and adapted to the syntax of each database, are provided in Supplementary Material File S3.

Reference lists of potentially eligible articles and included studies were additionally screened to identify relevant publications not captured by the electronic search. Records retrieved from the three databases were merged, and duplicate citations were identified and removed before title and abstract screening. No restrictions were applied with respect to language, geographic region, or clinical setting.

### 2.3. Eligibility Criteria

Studies were considered eligible if they enrolled adult participants (≥18 years) and provided original data relevant to either of the two prespecified domains of interest: (1) factors associated with asymptomatic AF or its detection/progression, and/or (2) clinical outcomes associated with asymptomatic AF. Eligible studies included patients with clinically diagnosed asymptomatic or silent AF, AF classified as asymptomatic according to symptom-based scales such as the European Heart Rhythm Association (EHRA) classification [31], and subclinical AF detected by prolonged electrocardiographic monitoring or cardiac implantable electronic devices. Studies directly comparing asymptomatic with symptomatic AF were eligible, as were studies using a non-AF or other study-specific comparator when they provided relevant information on factors associated with asymptomatic/subclinical AF or its subsequent progression. This broader approach was required because the included evidence encompassed conventional AF cohorts as well as screening and device-monitoring populations.

Eligible designs comprised prospective or retrospective observational cohorts, registries, cross-sectional and case–control studies, and relevant post hoc analyses of clinical trials. Studies were required to provide sufficient quantitative information on demographic, clinical, anthropometric, laboratory, electrocardiographic, imaging, or therapeutic correlates of asymptomatic AF and/or on clinically relevant endpoints, including mortality, thromboembolic events, myocardial infarction or acute coronary syndromes, HF, hospitalization, bleeding, AF progression, or composite cardiovascular outcomes.

Studies were excluded if they did not provide extractable data relevant to either review objective, did not permit identification of an asymptomatic/subclinical AF group, or consisted of reviews, meta-analyses, editorials, commentaries, case reports, conference abstracts without sufficient data, or non-original publications. When multiple reports originated from the same cohort, individual publications were retained only when they provided distinct and relevant information for the prespecified review objectives.

### 2.4. Study Selection

Records retrieved from the electronic searches were merged and duplicates removed before screening. Study selection was conducted in two stages according to the predefined eligibility criteria. Two investigators (A.S. and G.L.N.) independently performed study selection at both the title/abstract screening and full-text eligibility assessment stages. Titles and abstracts were initially screened to exclude clearly irrelevant or non-original publications and studies outside the scope of the review. Potentially eligible reports subsequently underwent full-text assessment based on study population, AF ascertainment and symptom status, comparator group, study design, and availability of quantitative data addressing at least one of the prespecified review objectives.

Full-text reports lacking relevant quantitative data or an identifiable asymptomatic/subclinical AF population were excluded. When potentially overlapping populations or multiple reports from the same registry were identified, studies were retained only when they provided distinct and complementary information on associated factors or clinical outcomes. Any disagreements regarding study eligibility or inclusion were resolved through discussion and consensus between the two investigators.

The selection process was documented according to the PRISMA 2020 framework. Reasons for full-text exclusion and reports that could not be retrieved were recorded, and the complete study-selection process is presented in the PRISMA flow diagram.

### 2.5. Data Extraction and Definition of Variables

Data were extracted using a predefined standardized spreadsheet specifically developed for the present systematic review. Data extraction was independently performed by two investigators (A.S. and G.L.N.), who subsequently cross-checked the extracted information for completeness and accuracy. Any discrepancies were resolved through discussion and consensus, with reference to the original publications. For each eligible study, the following information was collected when available: first author, country, year of publication, study design, characteristics of the study population, sample size, definition and method of ascertainment of asymptomatic AF, comparator group, and duration of follow-up. Data were extracted separately for asymptomatic and symptomatic AF whenever the original study provided a direct comparison; for studies using alternative reference populations, the study-specific comparator was retained.

Baseline variables encompassed demographic and anthropometric characteristics; cardiovascular risk factors and comorbidities; AF type and clinical presentation; heart rate and blood pressure; laboratory and circulating biomarkers; electrocardiographic variables; conventional and functional echocardiographic measurements; thromboembolic and bleeding risk scores; cardiac device use; and pharmacological and non-pharmacological AF management. Imaging variables included measures of cardiac chamber size and function, with particular attention to left atrial dimensions, volume, and functional indices, as well as ventricular structure and function when reported. Thoracic morphometric measurements were also extracted when available.

For the analysis of factors associated with asymptomatic AF, study-specific association measures were preferentially extracted from multivariable analyses and included odds ratios (ORs), adjusted odds ratios (aORs), regression coefficients, and their corresponding 95% confidence intervals (CIs) or *p* values, as reported by the original investigators. When adjusted estimates were unavailable, relevant unadjusted associations were retained and identified accordingly.

Clinical outcomes were extracted according to the definitions used in each individual study. Prespecified outcomes included all-cause and cardiovascular mortality, ischemic stroke, transient ischemic attack, systemic embolism, myocardial infarction or acute coronary syndrome, HF and HF hospitalization, overall and cardiovascular hospitalization, major or other clinically relevant bleeding, AF progression or recurrence, and composite cardiovascular or thromboembolic endpoints. Study-specific predictors of clinical outcomes were preferentially extracted from multivariable analyses and reported as hazard ratios (HRs), adjusted hazard ratios (aHRs), or other effect measures provided by the original investigators, together with their corresponding 95% CIs or *p* values. Follow-up duration and the comparator used for each outcome estimate were also recorded. No attempt was made to convert different effect measures into a common summary metric.

Because definitions of asymptomatic AF, AF subtype, clinical endpoints, and measurement techniques differed across studies, variables were harmonized only when they were considered clinically comparable. Original study definitions were otherwise retained, and no assumptions were made for variables that were not reported. Data unavailable in the source publication were recorded as missing rather than imputed.

### 2.6. Statistical Analysis

Given the substantial clinical and methodological heterogeneity across the included studies, a formal meta-analysis was not performed. Quantitative data were instead synthesized using descriptive and exploratory methods. For each variable, the number of contributing studies and the total number of participants with available data were recorded separately for asymptomatic and symptomatic AF.

For continuous variables, study-level central estimates were combined using sample-size weighting. The resulting weighted estimate was accompanied by the range of the central estimates reported across contributing studies. No conversion of interquartile ranges into estimated standard deviations was performed, as the descriptive synthesis did not require harmonization of dispersion measures for inferential statistical testing. Whenever available, the original study-specific measures of dispersion were retained without transformation. For variables available from a single study, the original study-specific estimate and corresponding dispersion were retained without further aggregation. Categorical variables were summarized by aggregating the reported numbers of events and denominators across studies and expressing the results as proportions.

Descriptive comparisons between asymptomatic and symptomatic AF were presented for demographic and clinical characteristics, AF phenotype, laboratory and biomarker variables, electrocardiographic and echocardiographic parameters, risk scores, device use, and therapeutic management. Because these comparisons were based on aggregated study-level data derived from heterogeneous populations, with asymptomatic and symptomatic estimates not necessarily originating from the same contributing studies, no inferential between-group statistical testing was performed. Accordingly, no aggregated *p* values were calculated, and all numerical differences were interpreted descriptively, without implying statistically significant associations or causal relationships.

Study-specific measures of association with asymptomatic AF and clinical outcomes were retained in the metric reported by the original investigators, including ORs, aORs, HRs, and aHRs, together with their corresponding 95% CIs. These estimates were displayed graphically on a logarithmic scale to facilitate comparison of their magnitude and direction. No statistical pooling across different effect measures, study populations, comparator groups, or follow-up intervals was undertaken. Accordingly, the forest plots provide a structured visual synthesis of the available study-specific evidence and should not be interpreted as conventional meta-analytic forest plots with pooled summary estimates.

Descriptive data aggregation and graphical analyses were performed using Python (3.12.14) (Python Software Foundation), with standard scientific computing libraries. No inferential statistical tests were conducted on the aggregated data, and no thresholds for statistical significance were applied to the descriptive comparisons.

### 2.7. Methodological Quality Assessment

Methodological quality and risk of bias were assessed using design-specific critical appraisal instruments developed by the Joanna Briggs Institute (JBI) [34]. The JBI checklist for cohort studies was applied to prospective and retrospective longitudinal observational studies, including registry-based investigations and observational secondary analyses of randomized trials. The JBI checklists for analytical cross-sectional and case–control studies were used when appropriate to the actual study design.

Particular attention was given to participant selection, ascertainment of AF and symptom status, identification and management of potential confounders, validity and reliability of exposure and outcome measurements, completeness of follow-up, and appropriateness of statistical analyses. For studies derived from randomized trials, the methodological assessment was based on the observational comparisons relevant to the present review rather than on the randomized design of the parent trial.

Methodological quality was evaluated independently by two investigators (A.S. and G.L.N.), and disagreements were resolved through discussion and consensus. Individual methodological domains were assessed qualitatively, without calculating numerical summary scores or applying arbitrary thresholds to classify study quality. Particular methodological concerns were considered when interpreting study-specific findings and the overall consistency of the available evidence.

## 3. Results

### 3.1. Study Selection and Characteristics of the Evidence Base

The database searches retrieved 41,412 records, comprising 8173 from PubMed, 12,865 from Scopus, and 20,374 from EMBASE. Based on the deduplication analysis of the available bibliographic records, an estimated 17,017 duplicate records were identified, leaving approximately 24,395 records for title and abstract screening. During screening, 24,307 records were excluded, and 88 reports were considered potentially eligible and sought for full-text retrieval. Of these, 10 could not be retrieved despite additional attempts through institutional bibliographic services. The bibliographic details of these 10 reports are provided in Supplementary Material File S4 to ensure transparency regarding potentially relevant evidence that could not be assessed for eligibility. Seventy-eight full-text reports were subsequently assessed, of which 55 were excluded because they did not provide sufficient information on factors associated with asymptomatic AF and/or relevant clinical outcomes. The final qualitative synthesis therefore comprised 23 studies [24,27,35,36,37,38,39,40,41,42,43,44,45,46,47,48,49,50,51,52,53,54,55] (Figure 1).

The 23 studies were published between 2013 and 2026 and encompassed geographically diverse populations from Europe, Asia, the Middle East, and North America, as well as large multinational cohorts. Collectively, the included datasets comprised 47,456 patients with asymptomatic or subclinical AF and 126,894 participants in the corresponding comparator groups, which included symptomatic AF patients, individuals without detected AF, and other study-specific reference populations. These overall sample sizes describe the entire evidence base and should not be interpreted as representing two directly comparable groups. The number of contributing studies and available participants varied according to the specific variable or outcome analyzed. The evidence base was predominantly observational and prospective, including single- and multicenter registries, community-based cohorts, hospital-based populations, and dedicated analyses of patients undergoing continuous rhythm monitoring. A smaller number of studies used retrospective, cross-sectional, case–control, or post hoc designs. Study populations were similarly heterogeneous, ranging from unselected or newly diagnosed AF cohorts to patients with persistent AF, acute myocardial infarction, hypertension, or implanted cardiac electronic devices. Accordingly, asymptomatic AF was identified through different approaches, including clinical symptom assessment, EHRA classification, conventional ECG, prolonged monitoring, and implanted devices. Sample size also varied markedly, from small mechanistic or imaging-oriented cohorts to large national and multinational registries comprising several tens of thousands of patients. Most studies directly compared asymptomatic with symptomatic AF, whereas selected investigations evaluated asymptomatic or subclinical AF against participants without detected AF or other study-specific comparator groups. Consequently, the descriptive summaries presented in the following sections reflect variable-specific data availability and should not be interpreted as paired between-group comparisons or pooled comparative effect estimates. The main design features, study populations, and comparator definitions are detailed in Table 1.

### 3.2. Clinical Phenotype Associated with Asymptomatic AF

Across the available datasets, the descriptive clinical characteristics of patients with asymptomatic AF showed heterogeneous numerical patterns across demographic, anthropometric, clinical, and arrhythmia-related variables (Table 2). These summaries reflect the characteristics of the contributing populations and should not be interpreted as evidence of a distinct clinical phenotype independently associated with asymptomatic AF.

Descriptive summaries of the available study-level data showed a slightly higher sample-size-weighted age estimate in the asymptomatic AF datasets and a numerically higher aggregated proportion of men (63.21% vs. 55.39%). Because these estimates were not necessarily derived from identical sets of studies, they should not be interpreted as direct between-group differences. Thoracic morphology was evaluated in a single study [24], in which asymptomatic AF was associated with a larger antero-posterior (A-P) thoracic diameter (17.4 vs. 14.3 cm) and a lower modified Haller index (MHI) (2.00 vs. 2.40). Across the broader study population, the descriptive summaries showed numerically lower resting heart rate estimates and a higher aggregated proportion of long-standing persistent or permanent AF in the asymptomatic datasets, while the reported proportions of first-diagnosed, paroxysmal, and persistent AF were relatively similar across the available datasets.

The clinical risk profile showed additional numerical differences in the descriptive summaries. Hypertension, diabetes, coronary artery disease (CAD), previous stroke/transient ischemic attack (TIA), peripheral artery disease (PAD), chronic kidney disease (CKD), chronic obstructive pulmonary disease (COPD), anemia, and previous bleeding showed higher aggregated proportions in the asymptomatic AF datasets, whereas HF showed a higher aggregated proportion in the symptomatic AF datasets. By contrast, body mass index (BMI), dyslipidemia, obesity, malignancy, and several less commonly reported comorbidities showed relatively similar descriptive estimates between groups. Notably, the number of contributing studies and participants varied considerably across individual variables, and several less frequently investigated characteristics were derived from only one or a few studies. Accordingly, these findings should be interpreted exclusively as descriptive summaries of heterogeneous study populations rather than as formal pooled estimates or direct inferential comparisons between asymptomatic and symptomatic AF. The observed numerical patterns may reflect differences in study composition, patient selection, AF ascertainment, and variable-specific data availability, rather than true differences attributable to AF symptom status.

### 3.3. Structural, Functional, and Biological Profile

Laboratory, electrocardiographic, and echocardiographic characteristics reported across the available asymptomatic and symptomatic AF datasets are descriptively summarized in Table 3. The estimates reflect variable-specific data availability and do not necessarily originate from directly comparable study populations.

Most conventional laboratory parameters showed relatively similar descriptive estimates across the available asymptomatic and symptomatic AF datasets, including hemoglobin, renal function, lipid profile, glucose, and N-terminal pro-B-type natriuretic peptide (NT-proBNP). Conversely, numerical differences were observed for selected biomarkers, with higher homocysteine, uric acid, and troponin I levels and lower high-sensitivity C-reactive protein (hs-CRP) values reported in the asymptomatic AF datasets. QRS duration showed a slightly higher descriptive estimate in asymptomatic AF, whereas electrocardiogram (ECG)-defined left ventricular (LV) hypertrophy showed a lower aggregated proportion.

Conventional measures of left ventricular (LV) structure and systolic function showed relatively similar descriptive estimates across the available symptom-status datasets. Interventricular septal (IVS) and posterior wall thickness, LV end-diastolic diameter, LV mass index, E/e′ ratio, and LV ejection fraction (LVEF) displayed no marked numerical differences in the aggregated summaries. In contrast, more pronounced numerical differences were observed in selected indices of atrial structure and function. The descriptive estimate of left atrial (LA) A-P diameter was higher in the asymptomatic AF datasets than in the symptomatic AF datasets (43.28 vs. 41.53 mm), while LA volume index (LAVi) showed relatively similar values. Available strain data showed numerically lower LA reservoir strain and left atrial appendage (LAA) global peak strain in the asymptomatic AF datasets, although these measurements were reported by only a small number of studies.

Selected right-sided and valvular parameters also showed numerical differences in the descriptive summaries, but the evidence was sparse. Mitral regurgitation showed a lower aggregated proportion in asymptomatic AF, whereas aortic regurgitation and stenosis also showed modest numerical differences between the available groups. Higher right ventricular (RV) inflow tract diameter and systolic pulmonary artery pressure (sPAP) and lower tricuspid annular plane systolic excursion (TAPSE) were reported in asymptomatic AF; however, these comparisons originated from a single study and therefore cannot be generalized to the broader AF population. Overall, these descriptive findings should be interpreted cautiously because of differences in contributing studies, population characteristics, and sample sizes, and should not be considered evidence of statistically significant between-group differences. In particular, the numerical patterns observed across the aggregated datasets cannot establish independent associations between AF symptom status and structural, functional, or biological characteristics. Such associations should instead be evaluated using the original study-specific effect estimates, accounting for their respective comparator populations and covariate adjustments.

### 3.4. Risk Scores and AF Management According to Symptom Status

Risk scores, cardiac device use, and therapeutic management are presented as descriptive summaries of the available study-level data in Table 4. The contributing studies and patient populations differed across individual parameters and symptom-status categories, limiting direct comparability of the aggregated estimates.

In the descriptive aggregated summaries, the asymptomatic AF datasets showed numerically higher CHA_2_DS_2_-VASc and HAS-BLED score estimates than the symptomatic AF datasets, although the absolute numerical differences were small and should not be interpreted as established differences in clinical risk according to symptom status. Permanent pacemaker implantation showed a lower aggregated proportion in asymptomatic AF, whereas implantable cardioverter-defibrillator (ICD) prevalence showed relatively similar descriptive values between groups.

Numerical differences were also observed in the descriptive summaries of pharmacological treatment patterns. Anticoagulant and antiplatelet use showed higher aggregated proportions in the asymptomatic AF datasets, as did treatment with angiotensin-converting enzyme (ACE) inhibitors/angiotensin receptor blockers (ARBs). Conversely, digoxin and mineralocorticoid receptor antagonist use showed lower aggregated proportions in asymptomatic AF. Antiarrhythmic drugs showed a slightly higher aggregated proportion in asymptomatic patients (20.16% vs. 17.12%), while statin use was also numerically higher (37.72% vs. 36.47%). Beta-blockers, angiotensin receptor–neprilysin inhibitors (ARNIs), calcium-channel blockers, and diuretics showed relatively similar descriptive estimates across the available datasets.

Rhythm-control strategies showed a lower aggregated proportion in asymptomatic than symptomatic AF datasets (39.63% vs. 56.88%), as did catheter ablation (12.02% vs. 15.31%). Conversely, electrical cardioversion showed a higher aggregated proportion in asymptomatic patients (9.78% vs. 4.12%). Given the variable number and size of contributing studies and the heterogeneity of the underlying populations, these numerical differences should be interpreted exclusively as descriptive findings rather than statistically established between-group differences. Furthermore, because the contributing studies were not necessarily identical across symptom-status groups, these estimates should not be considered direct comparative measures of treatment utilization. Rather, they describe numerical patterns of therapeutic management across the available datasets and cannot establish whether AF symptom status independently influenced treatment selection or utilization.

### 3.5. Device-Detected Subclinical AF and Continuously Monitored Silent AF

Seven studies specifically investigated asymptomatic or subclinical AF identified through implantable cardiac devices or continuous external ECG monitoring [38,40,43,45,47,49,52] (Table 5).

Importantly, clinically documented asymptomatic AF, device-detected subclinical AF/atrial high-rate episodes (AHRE), and screening-detected AF were considered distinct phenotypes because of differences in arrhythmia ascertainment, AF burden, comparator populations, treatment implications, and prognosis. Screening-detected AF, identified through conventional ECG or opportunistic clinical assessment, was therefore distinguished from device-detected subclinical AF, although the heterogeneity of the available evidence precluded separate quantitative analyses.

Among device-based investigations, Witkowski et al. [40] and Ahluwalia et al. [52] identified lower ventricular rates and shorter episode duration as relevant correlates of asymptomatic presentation. Wong et al. [43] demonstrated an association between SCAF progression and subsequent HF hospitalization, whereas Bertelsen et al. [45] identified LA enlargement and impaired atrial mechanical function as predictors of incident AF detected through continuous monitoring. Wang et al. [47] reported independent associations between circulating homocysteine and uric acid concentrations and SCAF occurrence. Additional studies using external ECG monitoring identified adverse in-hospital outcomes associated with silent AF following acute myocardial infarction [38] and an association between morning blood pressure surge and asymptomatic paroxysmal AF in hypertensive patients [49]. These findings highlight the heterogeneity of continuously monitored and device-detected AF populations, particularly regarding arrhythmia burden, comparator groups, and clinical outcomes. Accordingly, their results were synthesized descriptively without statistical pooling or direct cross-population comparisons. The available evidence was therefore interpreted according to the specific AF detection modality and study population, without assuming equivalence between clinical AF, device-detected SCAF, and screening-detected AF.

### 3.6. Factors Associated with Asymptomatic AF

Study-specific effect estimates identified several demographic, clinical, structural, and treatment-related factors associated with asymptomatic AF. Male sex emerged repeatedly across different populations, while older age was associated with asymptomatic AF in selected cohorts. AF phenotype and ventricular response also emerged as important correlates of symptom status. Non-paroxysmal AF, particularly persistent or permanent AF, and lower ventricular rates were consistently associated with asymptomatic AF. Associations with cardiovascular and systemic factors were less consistent across studies, although diabetes, previous stroke/TIA, PAD, prior bleeding, CAD, CKD, and greater overall comorbidity burden were associated with asymptomatic AF in selected cohorts. Conversely, HF, cardiomyopathy, and selected rhythm-control therapies were associated with a lower likelihood of asymptomatic AF in some populations (Figure 2).

Structural and functional markers further characterized asymptomatic AF (Figure 3). Greater LA size and adverse indices of LA remodeling—including higher maximal and minimal LA volumes, impaired emptying fractions, and reduced reservoir and active strain—were associated with asymptomatic or subclinical AF. Higher homocysteine and uric acid concentrations, particularly when both were elevated, were also associated with subclinical AF. Additional study-specific correlates included higher CHA_2_DS_2_-VASc and Charlson Comorbidity Index scores, whereas a higher MHI was inversely associated with asymptomatic AF. Across device-detected and subclinical AF cohorts, increasing age, BMI, and episode duration were associated with AF occurrence or progression. The magnitude and direction of these associations varied according to study population, AF definition, comparator group, and effect measure; therefore, the estimates displayed in Figure 2 and Figure 3 represent individual study associations rather than a pooled effect estimate.

### 3.7. Clinical Outcomes Associated with Asymptomatic AF

Clinical outcomes varied across populations, clinical settings, and follow-up durations. Several studies reported a higher risk of all-cause mortality in asymptomatic AF, with significant associations observed during hospitalization, at 1 year, and over longer follow-up; however, other large cohorts showed no significant difference in mortality between asymptomatic and symptomatic patients. A similarly heterogeneous pattern was observed for thromboembolic events. Increased risks of stroke or systemic embolism were reported in selected studies, whereas several contemporary cohorts showed comparable risks between symptom groups. Myocardial infarction and acute coronary syndrome were more frequent among asymptomatic patients in some settings, while estimates for composite cardiovascular outcomes varied in both magnitude and direction (Figure 4).

HF-related outcomes varied across study populations and clinical settings. In direct comparisons between asymptomatic and symptomatic AF, HF events and hospitalizations were generally more frequent among symptomatic patients. Conversely, an increased risk of HF hospitalization was observed in specific settings, particularly during progression of subclinical AF and among patients with concomitant HF and reduced LVEF.

AF progression to permanent AF was significantly increased during long-term follow-up in one cohort, while AF-related and overall cardiovascular hospitalizations were lower in other comparisons. Major bleeding was generally similar between asymptomatic and symptomatic AF, and vascular death did not differ significantly in the available analyses. Several studies additionally reported no significant differences in composite cardiovascular endpoints, stroke, mortality, or other major clinical events over intermediate- or long-term follow-up. The individual study estimates, comparator groups, and follow-up periods are detailed in Figure 5.

### 3.8. Methodological Appraisal of the Included Evidence

The methodological appraisal of the 23 included studies [24,27,35,36,37,38,39,40,41,42,43,44,45,46,47,48,49,50,51,52,53,54,55] was structured according to their respective study designs using the appropriate JBI critical appraisal instruments (Table 6). The detailed domain-level assessments for each study, including the individual JBI checklist judgments, are provided in Supplementary Material File S5 [24,27,35,36,37,38,39,40,41,42,43,44,45,46,47,48,49,50,51,52,53,54,55].

Rather than relying on numerical quality scores, the assessment focused on individual methodological domains potentially affecting the validity and interpretation of study findings.

The evidence base consisted predominantly of observational studies, including prospective registries, longitudinal cohorts, retrospective investigations, and cross-sectional analyses. Important methodological considerations included heterogeneity in AF ascertainment and symptom classification, potential selection bias, residual confounding, differences in follow-up duration, and variability in outcome assessment.

Studies involving continuous rhythm monitoring generally provided more detailed information on AF detection and episode characteristics, although their findings were influenced by the characteristics of the selected device-monitoring populations. Conversely, large observational registries offered broader clinical and prognostic information but remained susceptible to confounding and differences in symptom assessment.

These methodological considerations were incorporated into the qualitative interpretation of the evidence. No numerical quality scores were calculated, and no formal quantitative synthesis was undertaken.

## 4. Discussion

### 4.1. Principal Findings and Integrated Interpretation

This systematic review provides an integrated appraisal of the clinical phenotype, associated factors, management patterns, and prognosis of asymptomatic AF across 23 studies encompassing markedly different clinical settings and methods of arrhythmia detection. Asymptomatic AF did not correspond to a uniformly benign or low-risk clinical profile. Across the available evidence, asymptomatic AF was frequently observed in older individuals and men, while study-specific analyses identified lower ventricular rates and a greater representation of long-standing persistent or permanent AF as relevant correlates of symptom status. Several cardiovascular risk factors and vascular comorbidities also showed numerically higher proportions in the descriptive aggregated summaries of asymptomatic AF, although these estimates were derived from heterogeneous and not necessarily identical sets of studies and should not be interpreted as direct comparative associations.

The clinical phenotype of asymptomatic AF also involved potential differences in cardiac structure and function. While conventional measures of LV geometry and systolic function showed relatively similar descriptive estimates across the available datasets, asymptomatic AF showed numerically greater LA A-P dimensions, while study-specific analyses identified associations with indices of LA remodeling, including larger atrial volumes and impaired reservoir and active function. Selected biomarkers, particularly homocysteine and uric acid, were also associated with subclinical AF in individual studies, whereas anthropometric and thoracic morphometric characteristics emerged as less extensively investigated but potentially relevant correlates. Notably, the available study evaluating thoracic configuration found a larger A-P thoracic diameter and lower MHI in asymptomatic AF, an observation that deserves further investigation given the limited evidence currently available.

The available evidence also suggested differences in therapeutic management according to AF symptom status, although their magnitude and consistency varied across study populations. Descriptive summaries showed numerically lower proportions of rhythm-control strategies and catheter ablation in asymptomatic AF datasets, alongside higher aggregated anticoagulant use. However, these observations should not be interpreted as pooled comparative treatment effects, particularly because the contributing studies and patient populations were not necessarily identical between symptom-status groups.

Outcome estimates remained heterogeneous: some studies identified increased risks of mortality, stroke/systemic embolism, myocardial infarction, HF events, or AF progression, whereas several large contemporary cohorts found no significant differences in major cardiovascular endpoints between asymptomatic and symptomatic AF. Thus, symptom absence and cardiovascular risk should not be regarded as interchangeable constructs. The collective evidence instead supports asymptomatic AF as a heterogeneous clinical phenotype in which symptom perception, arrhythmia characteristics, atrial remodeling, comorbidity burden, treatment exposure, and the context in which AF is detected may each contribute differently to the observed clinical course.

The interpretation of these findings should also be contextualized within previous meta-analyses and contemporary randomized evidence. Earlier meta-analyses by Sgreccia et al. [26] and Karakasis et al. [28] reported broadly comparable mortality and thromboembolic risks between symptomatic and asymptomatic AF, although differences emerged for HF events and AF progression. Consistently, the EAST-AFNET 4 trial demonstrated the cardiovascular benefits of early rhythm-control therapy in patients with recently diagnosed clinical AF, with comparable treatment effects irrespective of symptom status [56]. Conversely, ARTESiA [57] and NOAH-AFNET 6 [58] investigated anticoagulation in device-detected subclinical AF or atrial high-rate episodes, highlighting the need to distinguish these populations from clinically documented asymptomatic AF. ARTESiA demonstrated a reduction in stroke/systemic embolism with apixaban, accompanied by increased major bleeding [57], whereas NOAH-AFNET 6 did not demonstrate a significant cardiovascular benefit with edoxaban and identified increased safety risks [58]. Collectively, these findings emphasize that AF management should be guided by arrhythmia characteristics, thromboembolic and bleeding risk rather than symptom status alone.

Importantly, associations involving atrial mechanical remodeling, circulating biomarkers, and particularly thoracic geometry remain largely observational or supported by individual studies and should therefore be interpreted as hypothesis-generating rather than established causal relationships or validated clinical markers.

### 4.2. Pathophysiological Mechanisms Underlying Asymptomatic AF and Its Clinical Outcomes

The mechanisms underlying the absence of symptoms during AF are likely multifactorial and appear to reflect the complex interaction between arrhythmia-related haemodynamics, atrial remodelling, underlying cardiovascular substrate, and individual symptom perception rather than a distinct electrophysiological entity [59,60,61,62].

An especially consistent signal across the available evidence concerns the ventricular response. Continuous monitoring data showed that symptomatic AF episodes were characterized by substantially higher ventricular rates than asymptomatic episodes, together with greater beat-to-beat irregularity and longer episode duration [63,64]. In the implantable-monitor study by Ahluwalia et al. [52], mean heart rate during AF was approximately 15 bpm higher during symptomatic episodes (103 vs. 88 bpm), and both heart rate and rhythm irregularity remained independently associated with symptom manifestation. This observation provides a plausible haemodynamic basis for symptom perception: a faster and more irregular ventricular response shortens diastolic filling time, increases beat-to-beat variation in stroke volume, and compounds the loss of effective atrial contraction, thereby reducing cardiac output and increasing filling pressures. These effects may become particularly relevant during exertion or in patients with limited ventricular reserve and may manifest clinically as palpitations, dyspnoea, fatigue, exercise intolerance, or overt congestion. Conversely, a slower ventricular response may permit better haemodynamic adaptation to AF and therefore attenuate symptom perception despite persistence of the arrhythmia. This interpretation is consistent with established AF physiology, in which symptoms may arise from the combined effects of loss of atrioventricular synchrony, irregular ventricular filling, rapid ventricular response, and sympathetic activation [65].

This haemodynamic framework may also help explain one of the most intriguing findings emerging from the present review: HF was generally less strongly associated with asymptomatic AF than with symptomatic AF. Hospitalized asymptomatic patients had a lower prevalence of HF and reduced LVEF, and symptomatic AF was more closely associated with in-hospital HF events. Similarly, the recent prospective VITAL Rhythm analysis demonstrated a significantly greater subsequent HF risk in symptomatic than asymptomatic AF, leading the investigators to suggest that AF-related symptoms may represent an early marker of underlying myocardial dysfunction or haemodynamic intolerance to the arrhythmia [54]. Several complementary mechanisms may contribute. Patients with impaired systolic or diastolic reserve are less able to compensate for the loss of atrial contraction, shortened filling time, and irregular ventricular cycle lengths and may therefore develop dyspnoea and congestion at an earlier stage [66,67]. A higher ventricular rate in symptomatic AF could further increase myocardial oxygen demand, shorten diastole, impair ventricular filling, and, when sustained, contribute to tachycardia-mediated ventricular dysfunction [68,69,70]. Conversely, asymptomatic patients may more often maintain a haemodynamically tolerated ventricular response. The RACE analysis [37] additionally suggested that asymptomatic patients with preserved quality of life may remain more physically active and less sedentary, potentially limiting HF development or progression, although this mechanism remains hypothetical. Thus, the lower HF burden observed in asymptomatic AF should not necessarily be interpreted as a protective effect of absent symptoms; rather, symptoms may identify the subgroup in whom AF interacts most adversely with limited myocardial and haemodynamic reserve.

At the same time, asymptomatic AF appears paradoxically associated with a more advanced atrial substrate. Several studies identified persistent or permanent rather than paroxysmal AF, longer-standing disease, and greater LA remodelling as correlates of asymptomatic presentation. In CODE-AF [51], absence of symptoms was associated with greater LA size, whereas symptomatic status was independently associated with a smaller LA anteroposterior diameter. The authors proposed that asymptomatic patients may have had AF for longer before diagnosis, permitting progressive atrial remodelling through sustained elevation of LA pressure, ineffective atrial emptying, and the reciprocal process whereby AF promotes the substrate that subsequently sustains AF. In a continuous-monitoring study, increased LA maximal and, particularly, minimal volumes, reduced total and active emptying fractions, and impaired reservoir and active strain independently predicted incident AF, which was predominantly asymptomatic [45]. Functional atrial impairment may therefore precede or accompany structural enlargement, supporting the concept of an underlying atrial cardiomyopathy that may progress silently [21]. The association of asymptomatic AF with persistent/permanent patterns could consequently reflect both adaptation to chronic arrhythmia and progressive attenuation of rhythm perception, while the atrial substrate continues to deteriorate [71,72,73]. Recent electrophysiological evidence further highlights the complex relationship between atrial structural remodeling and local electrical characteristics. Granata et al. [74] investigated the anatomical determinants of left atrial electrogram voltage using integrated computed tomography and high-density electroanatomical mapping. The authors demonstrated that myocardial muscle thickness was positively associated with local electrogram voltage, whereas intramyocardial fat infiltration contributed to voltage attenuation. Notably, omnipolar mapping showed greater sensitivity to variations in atrial tissue composition than conventional bipolar mapping, including during rhythm instability. These findings suggest that atrial electrogram characteristics reflect not only electrical activity but also underlying structural heterogeneity. Although a direct relationship with AF symptom perception was not investigated, such observations provide additional mechanistic insights into the atrial substrate potentially underlying clinically silent AF and emphasize the importance of integrating anatomical, functional, and electrophysiological assessment in future studies.

Demographic and systemic factors may further modulate this phenotype. Evidence from the broader literature suggests that male sex [75], older age [76,77], higher BMI [78], elevated systolic blood pressure [79], diabetes [80], chronic kidney disease [81], previous stroke [82], and greater overall comorbidity burden [83] may be associated with an asymptomatic AF presentation or reduced symptom perception. Notably, among the studies included in the present systematic review, in the prospective VITAL Rhythm cohort, male sex, higher BMI, and elevated systolic blood pressure were more strongly associated with asymptomatic than symptomatic incident AF [54]. These associations may partly reflect differences in autonomic responsiveness, physical activity, perception of palpitations, and healthcare-seeking behaviour, but they may also indicate a substrate characterized by chronic pressure and metabolic loading that favours atrial remodelling without necessarily producing an abrupt symptomatic response [84]. In older and multimorbid patients, dyspnoea, fatigue, and reduced exercise capacity may additionally be attributed to coexisting conditions rather than to AF itself, thereby reducing recognition of the arrhythmia as the cause of symptoms [85].

Importantly, this apparently greater haemodynamic tolerance should not be equated with biological benignity. The present evidence indicates that stroke, systemic embolism, myocardial infarction, and mortality are frequently comparable between symptomatic and asymptomatic AF, while some cohorts identified even higher risks among asymptomatic patients. The large GARFIELD-AF registry found essentially identical adjusted risks of mortality, stroke/systemic embolism, and major bleeding according to symptom status, despite clear phenotypic differences between the groups [46]. Similarly, the prospective VITAL Rhythm cohort found no significant difference in the magnitude of MI or stroke risk according to symptoms [54]. This dissociation is pathophysiologically plausible because the principal determinants of thromboembolism—atrial stasis, endothelial dysfunction, atrial cardiomyopathy, systemic vascular risk, and prothrombotic substrate—are not dependent on whether the patient perceives the arrhythmia. Indeed, greater LA remodelling and a higher prevalence of hypertension, diabetes, previous stroke, vascular disease, or renal dysfunction in several asymptomatic cohorts may counterbalance any apparent haemodynamic advantage conferred by a slower ventricular response.

Finally, delayed recognition and differential treatment exposure may represent the crucial link between silent AF and adverse outcomes [86,87]. Symptoms prompt medical evaluation, whereas asymptomatic paroxysmal AF may remain undetected until stroke, acute coronary events, or another complication brings the patient to medical attention. Esato et al. [42] specifically proposed that the excess mortality observed in asymptomatic paroxysmal AF could result from greater difficulty detecting intermittent silent episodes, delaying appropriate AF management and anticoagulation. Consistently, several included studies showed less frequent use of rhythm-control strategies, antiarrhythmic drugs, catheter ablation, and, in selected populations, oral anticoagulation among asymptomatic patients. In VITAL Rhythm, asymptomatic patients with an indication for anticoagulation were significantly less likely to receive oral anticoagulation (OAC) and were also less frequently treated with rate-control therapy [54]. Conversely, GARFIELD-AF demonstrated that once anticoagulation was prescribed, its protective association with stroke/systemic embolism and mortality was comparable in asymptomatic and symptomatic patients [46].

Taken together, these observations suggest a clinically relevant dual phenotype: symptomatic AF may predominantly identify haemodynamic intolerance and vulnerability to HF, whereas asymptomatic AF may permit prolonged, clinically silent exposure to atrial remodelling and thromboembolic risk. Symptom status should therefore be regarded primarily as a marker of individual perception and haemodynamic response to AF, rather than as a surrogate for arrhythmia severity or cardiovascular risk.

### 4.3. Thoracic Geometry as a Potential Extracardiac Modulator of AF Symptom Perception

An additional and largely unexplored determinant of AF symptom perception may reside outside the heart itself. Thoracic geometry may influence how cardiac motion and haemodynamic changes occurring during AF are mechanically transmitted to the chest wall and ultimately perceived by the patient. In a study conducted by our research group and specifically designed to investigate this hypothesis in patients with persistent AF undergoing electrical cardioversion, asymptomatic patients had a substantially larger A-P thoracic diameter than symptomatic patients (17.4 ± 1.3 vs. 14.3 ± 1.0 cm), despite comparable latero-lateral dimensions [24]. Consequently, the MHI, calculated as the ratio between latero-lateral and A-P thoracic diameters [88], was markedly lower in asymptomatic AF (2.0 ± 0.1 vs. 2.4 ± 0.1). Importantly, MHI remained independently and inversely associated with asymptomatic status after accounting for thromboembolic risk and overall comorbidity burden (OR 0.76 per 0.01 increase, 95% CI 0.59–0.97), with an MHI < 2.2 providing the best discrimination of an asymptomatic presentation.

Figure 6 illustrates the assessment of thoracic geometry and highlights the marked difference in A-P thoracic diameter between patients with wider and narrower chest configurations.

These findings raise the intriguing possibility that the physical relationship between the heart and the surrounding thoracic cage contributes to interindividual variability in arrhythmia perception. A narrow A-P diameter and consequently more concave chest configuration bring the anterior chest wall into closer spatial relationship with the cardiac chambers. Such geometry could facilitate mechanical transmission of irregular cardiac contractions to the thoracic surface and enhance awareness of palpitations. Conversely, a larger A-P diameter produces a more circular transverse thoracic configuration, potentially increasing the spatial separation between the heart and anterior chest wall and attenuating perception of irregular cardiac motion. The original study additionally proposed that the increased symptom burden associated with a narrow chest might reflect several interacting mechanisms, including reduced cardiac chamber dimensions imposed by thoracic geometry, the greater prevalence of mitral valve prolapse/mitral regurgitation, and enhanced adrenergic activation [89]. This hypothesis is particularly interesting in light of the present review, in which a lower ventricular rate repeatedly characterized asymptomatic AF: mechanical attenuation related to thoracic geometry and a less pronounced haemodynamic stimulus related to slower ventricular response could conceivably act together to reduce AF perception.

The relationship between thoracic configuration and symptom status may also intersect with atrial remodeling. In the same cohort, asymptomatic patients with a larger A-P diameter displayed larger cardiac chambers, greater LA volume, higher filling pressures, and substantially impaired LA and LAA mechanical function despite perceiving fewer symptoms. Thus, a relative absence of symptoms may coexist with, rather than exclude, advanced structural and functional cardiac abnormalities. This observation provides a potentially important conceptual link between the thoracic phenotype and the broader findings of the present systematic review: greater anatomical accommodation of the heart within a wider A-P thoracic cavity might reduce mechanical symptom perception while allowing atrial enlargement and remodeling to progress clinically unnoticed. Conversely, a narrow thoracic configuration might amplify cardiac perception even in patients with comparatively less advanced remodeling. This interpretation remains hypothesis-generating, since the evidence currently derives from a small, single-center retrospective cohort and cannot establish causality. Nevertheless, it introduces thoracic geometry as a novel extracardiac component of the AF symptom phenotype and provides a rationale for prospective studies evaluating direct A-P thoracic diameter and MHI alongside ventricular rate, AF burden, LA remodeling, autonomic indices, and standardized measures of symptom perception.

Figure 7 summarizes the principal demographic and systemic correlates of asymptomatic AF, together with the potential cardiac and extracardiac determinants of symptom perception, including thoracic geometry, and their clinical implications.

### 4.4. Implications for Clinical Practice

The present findings have several practical implications for the detection and management of asymptomatic AF. Most importantly, absence of symptoms should not be interpreted as evidence of low clinical risk. Across the included studies, asymptomatic patients often carried substantial thromboembolic and comorbidity burdens, and their risks of stroke, systemic embolism, myocardial infarction, or mortality were frequently comparable with those of symptomatic patients. Accordingly, decisions regarding anticoagulation, cardiovascular risk-factor control, and follow-up intensity should remain driven by the underlying clinical risk profile rather than by symptom status alone.

The identification of a recognizable asymptomatic phenotype may also help refine screening strategies. Older age, male sex, lower ventricular rate, persistent or permanent AF, greater atrial remodeling, higher BMI, elevated systolic blood pressure, and selected vascular comorbidities repeatedly emerged as correlates of silent AF in different populations. In this context, assessment of left atrial reservoir strain (LASr) may provide complementary information on the underlying atrial substrate, as impaired LASr can identify atrial mechanical dysfunction before overt structural remodeling and has been associated with atrial fibrosis, thromboembolic risk, and adverse outcomes in AF [90,91]. This may be particularly relevant in asymptomatic patients, in whom symptom burden cannot reliably reflect AF burden or the severity of the underlying atrial substrate. Current ESC/EHRA consensus emphasizes that symptoms should not be used as a surrogate for AF burden and supports extended rhythm monitoring when accurate burden assessment is clinically relevant [92]. Patients displaying several of these features may therefore warrant a lower threshold for prolonged rhythm monitoring, particularly when AF would otherwise remain clinically unsuspected. This may be especially relevant in individuals with previous stroke/TIA, high CHA_2_DS_2_-VASc scores, or substantial comorbidity burden, in whom delayed recognition of AF could postpone initiation of preventive therapy.

The findings also reinforce the need to separate symptom control from prognostic management. Rhythm-control interventions were generally used less frequently in asymptomatic patients, reflecting the traditional symptom-oriented approach to AF treatment [93]. However, contemporary evidence suggests that clinically relevant outcomes are not necessarily determined by symptom burden, and asymptomatic patients may still benefit from appropriate anticoagulation, cardiovascular risk modification, and, when otherwise indicated, rhythm-control strategies. In parallel, the lower prevalence of HF and lower HF-event rates observed in several asymptomatic cohorts suggest that symptoms may help identify patients with poorer myocardial reserve or greater haemodynamic intolerance to AF. In symptomatic patients, therefore, dyspnoea, fatigue, or exercise limitation should prompt careful assessment of ventricular function, filling pressures, rate control, and coexisting cardiopulmonary disease rather than being attributed to AF alone.

A further practical consideration emerging from the present review is the potential value of thoracic conformation assessment. In the study specifically addressing this issue [24], a larger A-P thoracic diameter and a lower MHI were independently associated with asymptomatic persistent AF, whereas a narrower, more concave chest configuration was more frequently observed in symptomatic patients. Although this evidence remains preliminary and should not yet be used as a stand-alone screening criterion, the measurement is simple, noninvasive, radiation-free, and potentially obtainable during routine echocardiographic evaluation. Thoracic geometry may therefore represent an adjunctive phenotypic marker in patients in whom symptom perception appears discordant with arrhythmia burden or structural remodeling. In particular, assessment of direct A-P thoracic diameter or MHI could complement conventional clinical variables when estimating the likelihood of silent AF, especially in older men with high thromboembolic risk and multiple comorbidities. The original study suggested that patients with a more circular thoracic configuration and MHI < 2.2 had an increased probability of asymptomatic AF, whereas those with a more concave configuration were more likely to perceive palpitations or dyspnoea [24].

Collectively, these observations support a more comprehensive approach to AF in which symptom status is treated as only one component of the clinical phenotype. Clinical assessment should integrate arrhythmia pattern and ventricular response, thromboembolic and bleeding risk, comorbidity burden, atrial structure and mechanical function, including LASr when available, treatment exposure, and, potentially, extracardiac determinants of symptom perception such as thoracic geometry. Such an approach may improve recognition of silent AF, reduce therapeutic delays, and help identify patients in whom the absence of symptoms masks clinically relevant cardiovascular risk.

A proposed practical framework for the identification, assessment, management, and longitudinal follow-up of patients with asymptomatic AF is summarized in Figure 8.

### 4.5. Strengths and Limitations

The present systematic review has several strengths. It provides a broad and integrated assessment of asymptomatic AF by examining not only its demographic and clinical correlates, but also anthropometric, laboratory, electrocardiographic, structural, functional, and therapeutic characteristics together with subsequent clinical outcomes. The inclusion of 23 studies encompassing diverse geographical regions, healthcare settings, AF phenotypes, and methods of rhythm detection allowed the asymptomatic phenotype to be examined across a wide spectrum of contemporary AF populations. Particular attention was given to study-specific adjusted effect estimates, thereby distinguishing independent associations from purely descriptive differences. The review also incorporated emerging domains that have received comparatively limited attention, including LA mechanical function, circulating biomarkers, AF burden and episode characteristics, and thoracic morphology. Finally, the parallel evaluation of phenotype, management, and prognosis provides a clinically relevant framework for interpreting the absence of AF-related symptoms beyond symptom perception alone.

Several limitations should nevertheless be acknowledged. The most important is the substantial clinical and methodological heterogeneity of the available literature. Definitions of asymptomatic AF varied across studies and included physician-reported absence of symptoms, EHRA-based classifications [31], incidentally detected AF, and subclinical or device-detected episodes. These entities are related but not interchangeable, and differences in AF ascertainment may have influenced both the observed phenotype and subsequent outcomes. Study populations were similarly heterogeneous, ranging from community-based cohorts and large AF registries to hospitalized patients, individuals with persistent AF, patients after myocardial infarction, and recipients of implanted cardiac devices. Comparator groups, follow-up duration, endpoint definitions, and covariate adjustment also differed considerably.

The predominantly observational nature of the evidence precludes causal inference and leaves the possibility of residual and unmeasured confounding. Symptom classification is intrinsically subjective and may be influenced by age, sex, physical activity, comorbidities, psychological factors, individual perception, and attribution of nonspecific manifestations such as fatigue or dyspnoea. Moreover, symptom status was generally determined at a particular time point, although AF symptoms may change over time and within the same individual. Detection bias is also relevant: symptomatic patients are more likely to seek medical attention, whereas silent AF may remain undiagnosed until prolonged monitoring, incidental examination, or a cardiovascular complication leads to its recognition.

An additional limitation concerns the inability to retrieve 10 of the 88 reports sought for full-text assessment (11.4%), despite additional attempts to obtain these publications through institutional bibliographic services. Although their bibliographic details are provided in Supplementary Material File S4, their eligibility and potential contribution to the present review could not be determined. Therefore, availability bias cannot be excluded, as potentially relevant evidence may have been omitted from the final qualitative synthesis. This limitation should be considered when interpreting the completeness and generalizability of the findings.

Because of the marked heterogeneity in populations, definitions, effect measures, comparators, and follow-up intervals, a formal meta-analysis was not considered methodologically appropriate. The aggregated data presented in this review were therefore retained exclusively as descriptive summaries rather than pooled comparative effect estimates. Importantly, the asymptomatic and symptomatic AF estimates were not always derived from identical sets of studies, and some investigations included non-AF controls or other study-specific comparator populations. Consequently, direct inferential comparisons between the aggregated groups would have been methodologically inappropriate. To address this limitation, no between-group inferential statistical testing was performed on the aggregated data, and no corresponding *p* values were reported. Numerical differences between descriptive estimates should therefore not be interpreted as statistically established associations with AF symptom status. The number of contributing studies and participants varied substantially across individual variables, and some potentially relevant associations were supported by only one or a few investigations. Similarly, the forest plots were intended to provide a structured visual representation of study-specific estimates and should not be interpreted as conventional meta-analytic forest plots. Nevertheless, study-specific adjusted effect estimates and their corresponding confidence intervals were preserved to distinguish associations identified within individual investigations from the descriptive patterns observed across the broader evidence base.

Outcome interpretation requires particular caution. Differences in anticoagulation, rate- and rhythm-control strategies, catheter ablation, cardiovascular risk-factor management, and historical treatment standards may have modified the relationship between symptom status and prognosis. Furthermore, clinical AF and device-detected subclinical AF may represent different stages of the disease continuum, particularly with respect to AF burden and thromboembolic risk. The apparently lower burden of HF among asymptomatic patients should therefore not be interpreted as evidence of a protective effect of symptom absence, since symptoms themselves may identify underlying myocardial dysfunction or poorer haemodynamic tolerance to AF.

Finally, several of the most mechanistically interesting findings require external validation. Detailed indices of LA mechanical remodeling, circulating biomarkers, and thoracic morphology were evaluated in relatively limited populations. In particular, the association between a larger A-P thoracic diameter, lower MHI, and reduced AF symptom perception derives from a small, retrospective, single-center cohort of patients with persistent AF undergoing electrical cardioversion [24]. These findings should therefore be considered hypothesis-generating rather than immediately generalizable.

Prospective multicenter studies integrating standardized symptom assessment with continuous AF-burden quantification, ventricular rate, atrial structure and function, thoracic geometry, and longitudinal clinical outcomes will be needed to determine whether these emerging markers add incremental value to established clinical risk stratification.

## 5. Conclusions

Asymptomatic AF should not be considered a benign form of the arrhythmia. It represents a heterogeneous phenotype characterized by distinct demographic and clinical features, with study-specific evidence identifying lower ventricular rates, more sustained AF patterns, and greater atrial remodeling as relevant correlates of asymptomatic presentation. Thoracic conformation has also been proposed as a potential extracardiac correlate of AF symptom perception; however, this hypothesis is currently supported by a single small retrospective study and requires independent validation.

Despite a lower burden of HF events reported among asymptomatic patients in several individual studies, asymptomatic patients may remain exposed to substantial thromboembolic and cardiovascular risk, with mortality and major vascular outcomes frequently comparable to those of symptomatic AF. Thus, the absence of symptoms should not be used to reduce the intensity of indicated cardiovascular prevention or replace established risk stratification.

A comprehensive approach integrating AF pattern, ventricular rate, comorbidities, atrial remodeling, and other established clinical determinants may improve recognition and management of silent AF. However, the available evidence remains heterogeneous, and descriptive differences across study populations should not be interpreted as independently established associations. The potential contribution of extracardiac factors, including thoracic geometry, remains exploratory and should not currently influence clinical decision-making. Prospective studies are needed to refine phenotypic characterization and identify patients who may benefit most from targeted screening and earlier intervention.

## Figures and Tables

**Figure 1:**
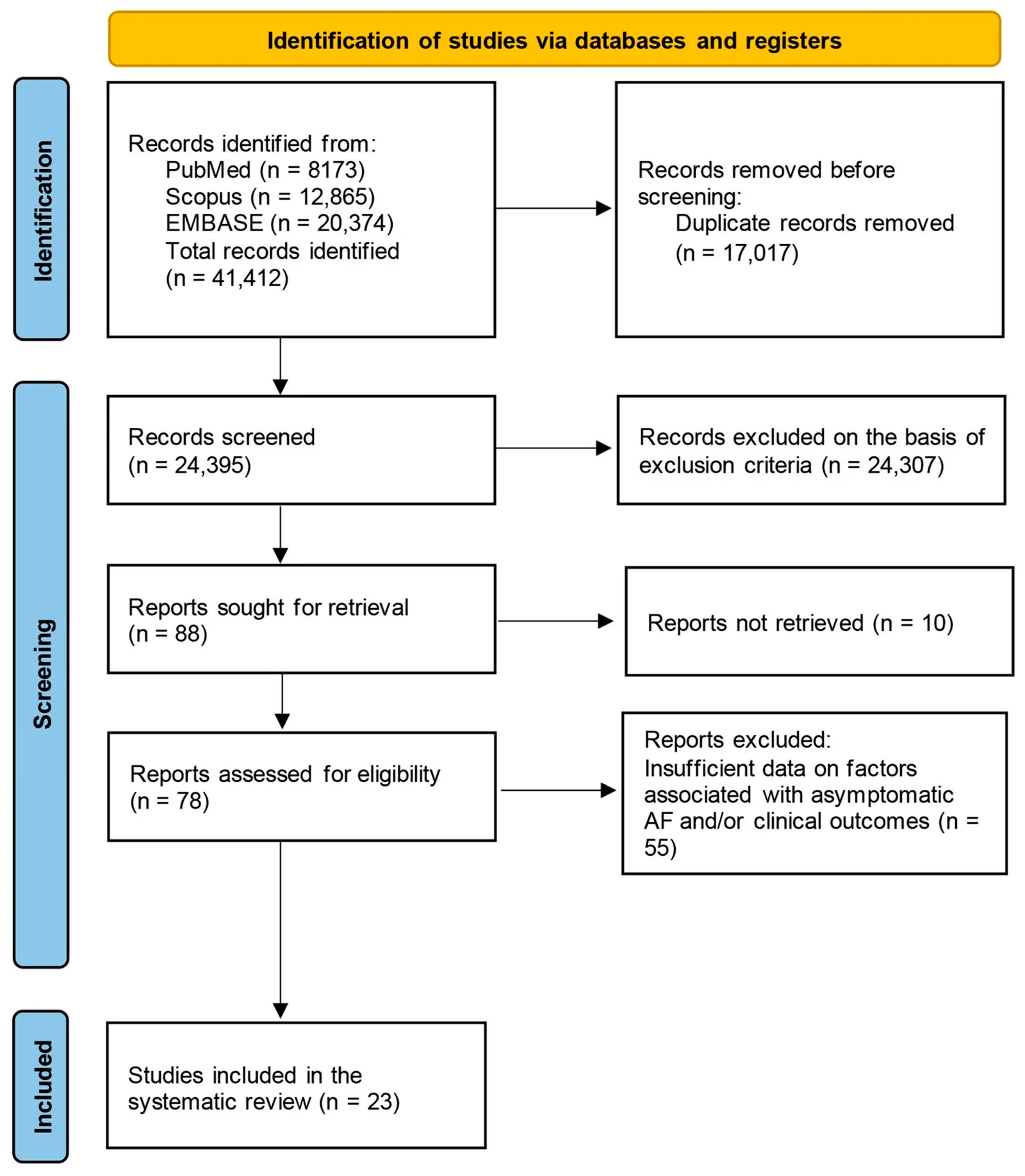
PRISMA 2020 flow diagram illustrating the identification, screening, eligibility assessment, and final inclusion of studies in the systematic review. AF, atrial fibrillation; PRISMA, Preferred Reporting Items for Systematic Reviews and Meta-Analyses.

**Figure 2:**
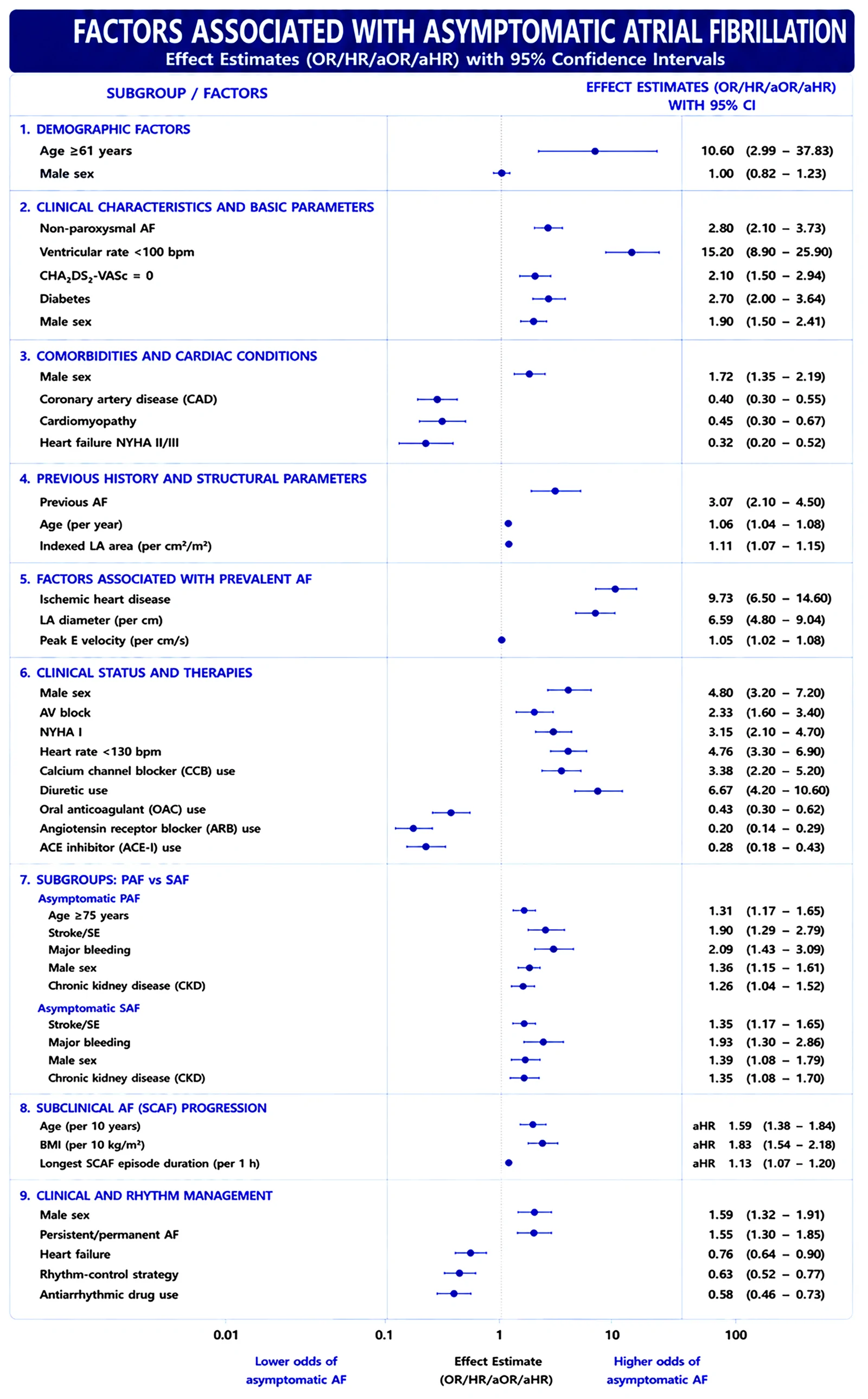
Factors Associated With Asymptomatic Atrial Fibrillation. Study-specific effect estimates for demographic, clinical, cardiovascular, structural, and treatment-related factors associated with asymptomatic AF, including subgroup analyses according to AF phenotype and subclinical AF progression. Points represent effect estimates and horizontal lines indicate 95% confidence intervals. Estimates are displayed on a logarithmic scale. Values < 1 indicate a lower likelihood of asymptomatic AF and values > 1 a higher likelihood. Effect estimates are reported as provided by the original studies and should not be interpreted as pooled meta-analytic estimates. ACE-i, angiotensin-converting enzyme inhibitor; AF, atrial fibrillation; aHR, adjusted hazard ratio; aOR, adjusted odds ratio; ARB, angiotensin receptor blocker; AV, atrioventricular; BMI, body mass index; CAD, coronary artery disease; CCB, calcium-channel blocker; CHA_2_DS_2_-VASc, congestive heart failure, hypertension, age ≥ 75 years, diabetes mellitus, stroke/transient ischemic attack/thromboembolism, vascular disease, age 65–74 years, and sex category; CI, confidence interval; CKD, chronic kidney disease; HR, hazard ratio; LA, left atrial; NYHA, New York Heart Association; OAC, oral anticoagulant; OR, odds ratio; PAF, paroxysmal atrial fibrillation; SAF, sustained atrial fibrillation; SCAF, subclinical atrial fibrillation; SE, systemic embolism.

**Figure 3:**
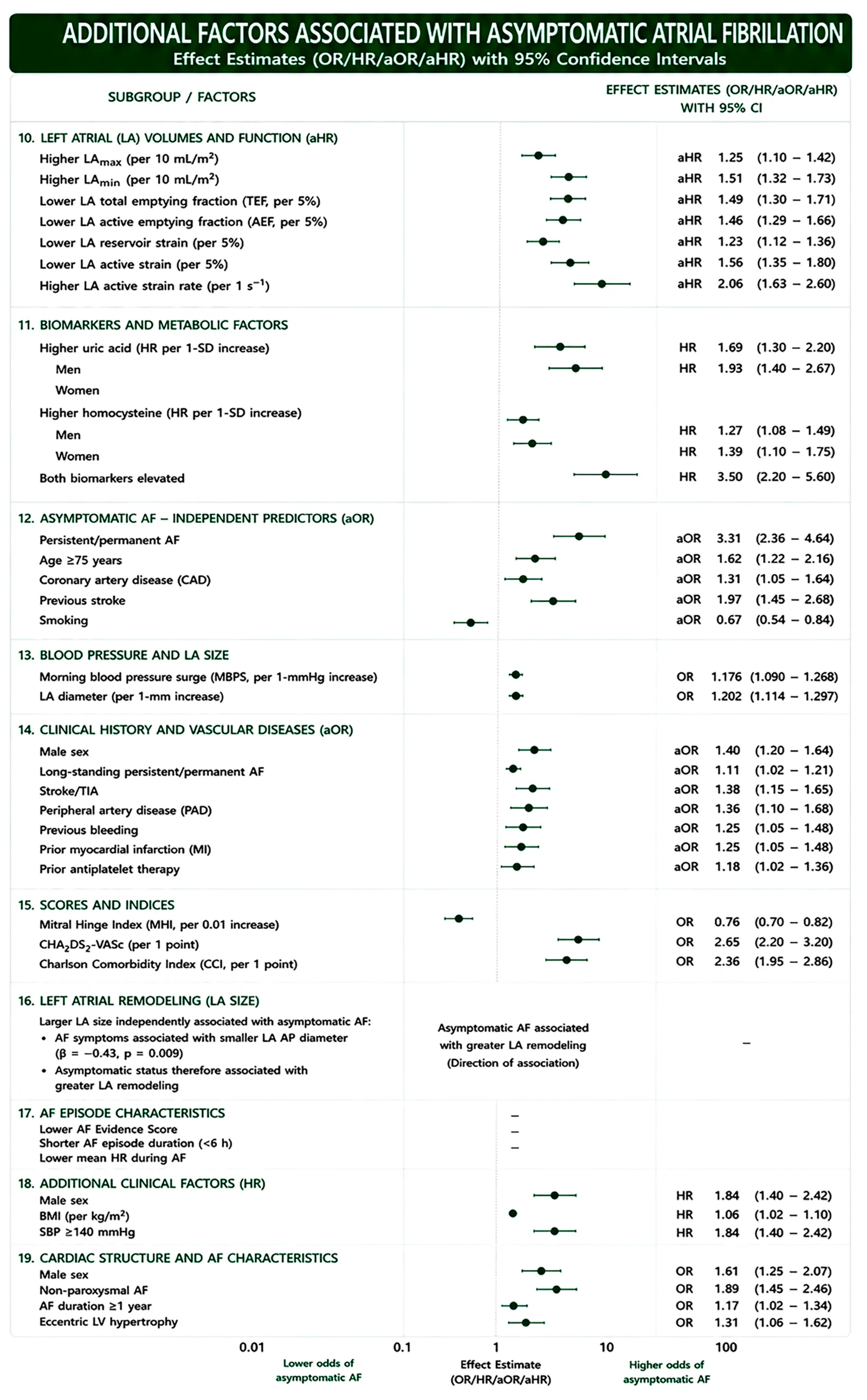
Additional Factors Associated With Asymptomatic Atrial Fibrillation. Study-specific associations of left atrial structure and function, biomarkers, metabolic and vascular factors, comorbidity indices, thoracic morphology, AF episode characteristics, and additional clinical and cardiac features with asymptomatic AF. Points and horizontal lines represent effect estimates and 95% confidence intervals, respectively, on a logarithmic scale. Where numerical effect estimates were unavailable, the direction of the reported association is shown. Estimates retain the effect measure and unit reported in the original study and represent individual study findings rather than pooled estimates. Adjusted estimates (aORs and aHRs) are distinguished from unadjusted estimates (ORs and HRs) by their corresponding labels. These effect measures are not directly interchangeable, and their graphical presentation is intended solely to illustrate the direction and magnitude of study-specific associations. Findings derived from device-detected SCAF populations should be interpreted separately from those involving clinically documented asymptomatic AF, given differences in arrhythmia ascertainment, comparator groups, and clinical characteristics. AEF, active emptying fraction; AF, atrial fibrillation; aHR, adjusted hazard ratio; aOR, adjusted odds ratio; BMI, body mass index; CAD, coronary artery disease; CCI, Charlson Comorbidity Index; CHA_2_DS_2_-VASc, congestive heart failure, hypertension, age ≥ 75 years, diabetes mellitus, stroke/transient ischemic attack/thromboembolism, vascular disease, age 65–74 years, and sex category; CI, confidence interval; HR, hazard ratio; LA, left atrial; LV, left ventricular; MHI, modified Haller index; MI, myocardial infarction; OR, odds ratio; SBP, systolic blood pressure; SCAF, subclinical atrial fibrillation; TEF, total emptying fraction; TIA, transient ischemic attack.

**Figure 4:**
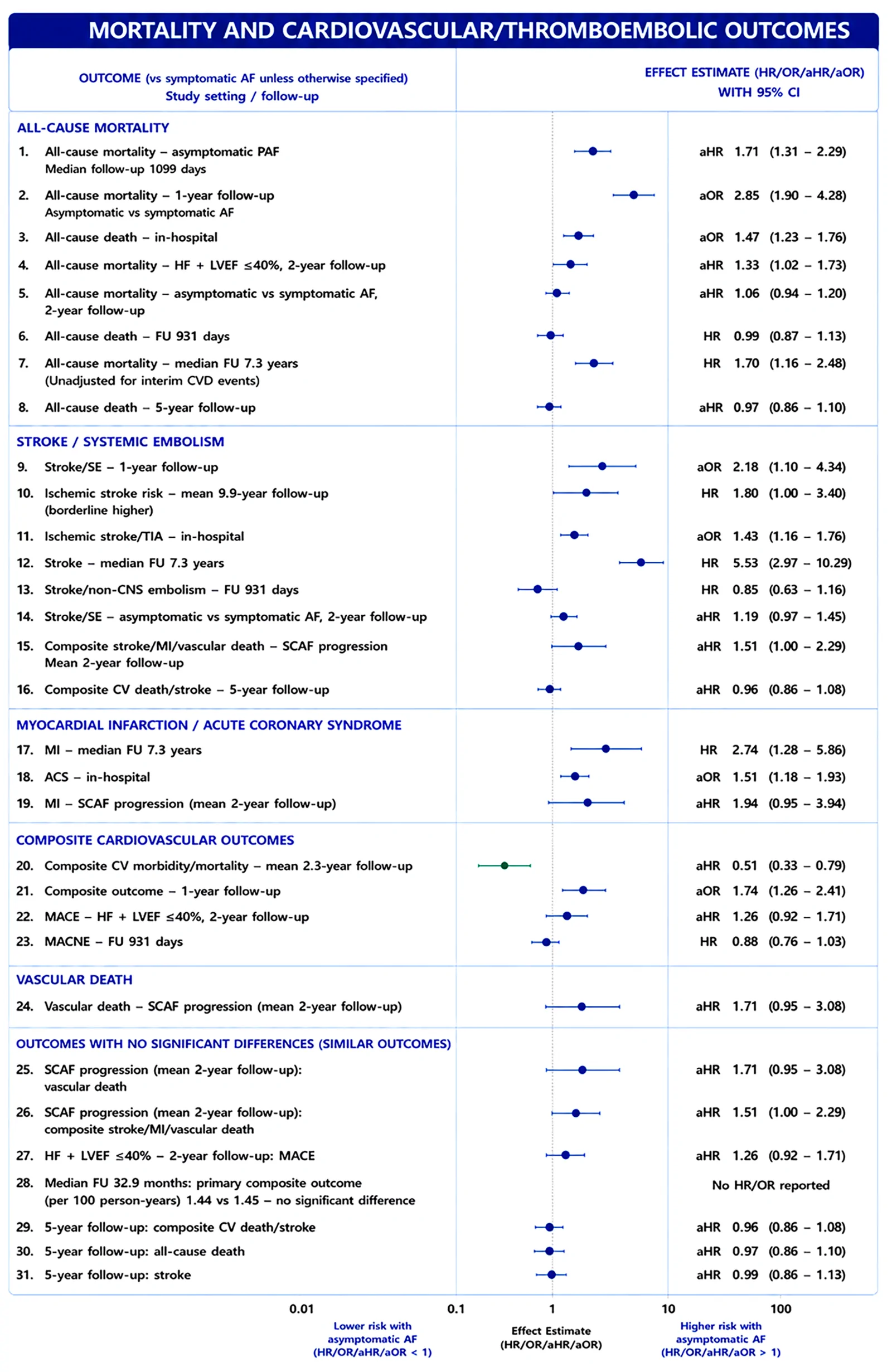
Mortality and Cardiovascular and Thromboembolic Outcomes Associated With Asymptomatic Atrial Fibrillation. Study-specific effect estimates for all-cause mortality, stroke/systemic embolism, myocardial infarction/acute coronary syndrome, composite cardiovascular outcomes, and vascular death in patients with asymptomatic AF compared with symptomatic AF unless otherwise specified. Points represent effect estimates and horizontal lines indicate 95% confidence intervals on a logarithmic scale. Values below 1 indicate lower risk and values above 1 higher risk associated with asymptomatic AF only when the comparison is asymptomatic versus symptomatic AF; for other comparisons, the direction of association must be interpreted according to the study-specific reference group. Effect measures, comparator groups, and follow-up durations are retained as reported in the original studies. No pooled summary estimate was calculated. Adjusted estimates (aORs and aHRs) and unadjusted estimates (ORs and HRs) are identified by their respective labels and should not be considered directly comparable. Results concerning device-detected SCAF progression are presented alongside clinical AF outcomes for descriptive purposes only and should be interpreted separately, particularly because the reference populations and endpoints differ. ACS, acute coronary syndrome; AF, atrial fibrillation; aHR, adjusted hazard ratio; aOR, adjusted odds ratio; CI, confidence interval; CNS, central nervous system; CV, cardiovascular; CVD, cardiovascular disease; FU, follow-up; HF, heart failure; HR, hazard ratio; LVEF, left ventricular ejection fraction; MACE, major adverse cardiovascular events; MACNE, major adverse cardiovascular and neurological events; MI, myocardial infarction; OR, odds ratio; PAF, paroxysmal atrial fibrillation; SCAF, subclinical atrial fibrillation; SE, systemic embolism; TIA, transient ischemic attack.

**Figure 5:**
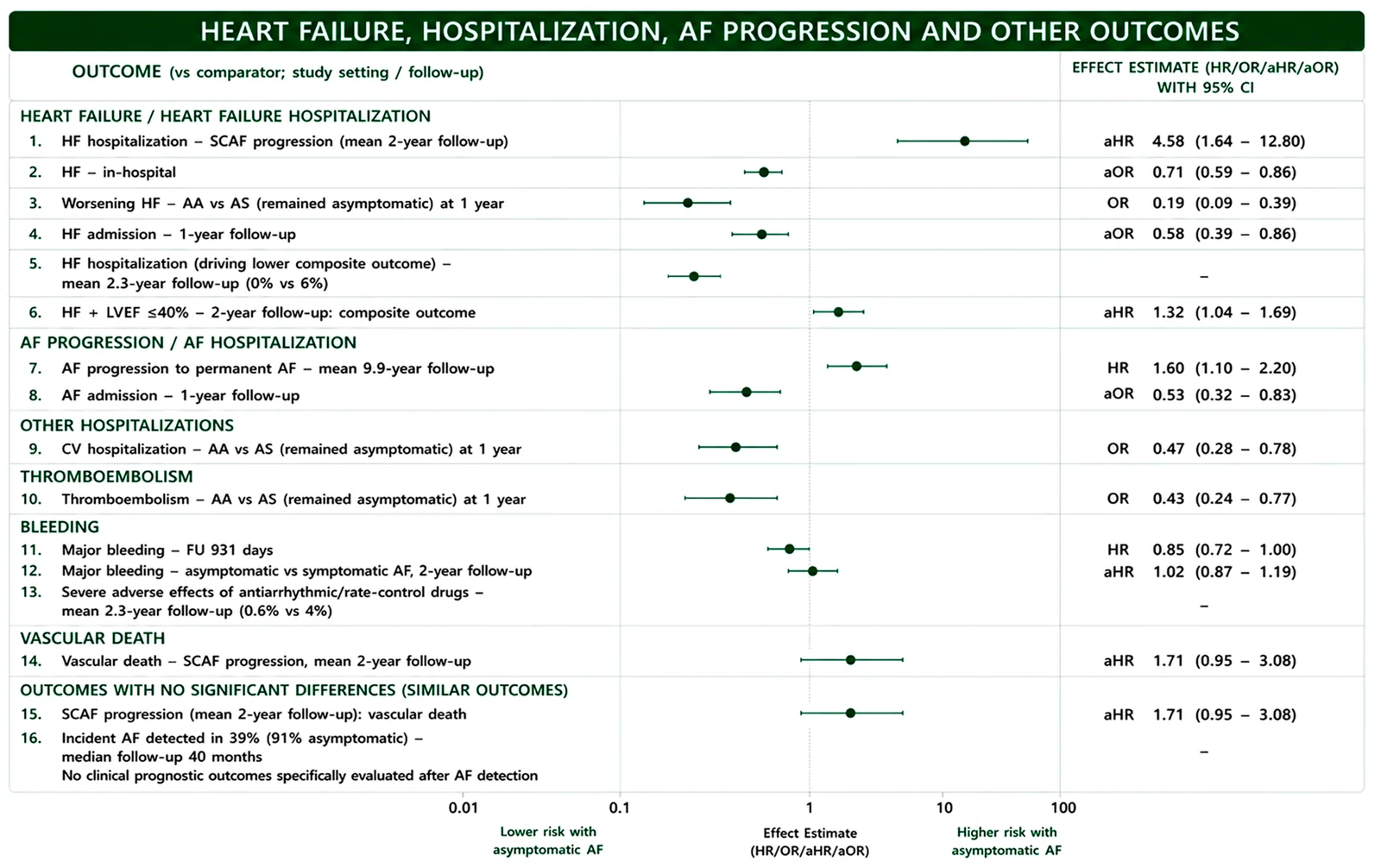
Heart Failure, Hospitalization, Atrial Fibrillation Progression, and Other Clinical Outcomes. Study-specific findings for heart failure and heart failure hospitalization, AF progression and hospitalization, cardiovascular hospitalization, thromboembolism, bleeding, vascular death, and other reported outcomes associated with asymptomatic AF. Points and horizontal lines represent effect estimates and 95% confidence intervals, respectively, on a logarithmic scale. Values below 1 indicate lower risk and values above 1 higher risk associated with asymptomatic AF according to the study-specific comparator. For outcomes involving SCAF progression or alternative reference populations, the direction of association should be interpreted according to the original study comparison rather than symptom status alone. Outcomes for which no effect estimate was available are presented descriptively. No pooled summary estimate was calculated. Adjusted (aORs and aHRs) and unadjusted (ORs and HRs) estimates are explicitly identified by their labels and should not be interpreted as equivalent measures. Device-detected SCAF and clinically documented asymptomatic AF represent distinct populations; therefore, their findings are displayed for structured descriptive comparison only, without statistical pooling or direct cross-population inference. AA, asymptomatic atrial fibrillation; AF, atrial fibrillation; aHR, adjusted hazard ratio; aOR, adjusted odds ratio; AS, symptomatic atrial fibrillation; CI, confidence interval; CV, cardiovascular; FU, follow-up; HF, heart failure; HR, hazard ratio; LVEF, left ventricular ejection fraction; OR, odds ratio; SCAF, subclinical atrial fibrillation.

**Figure 6:**
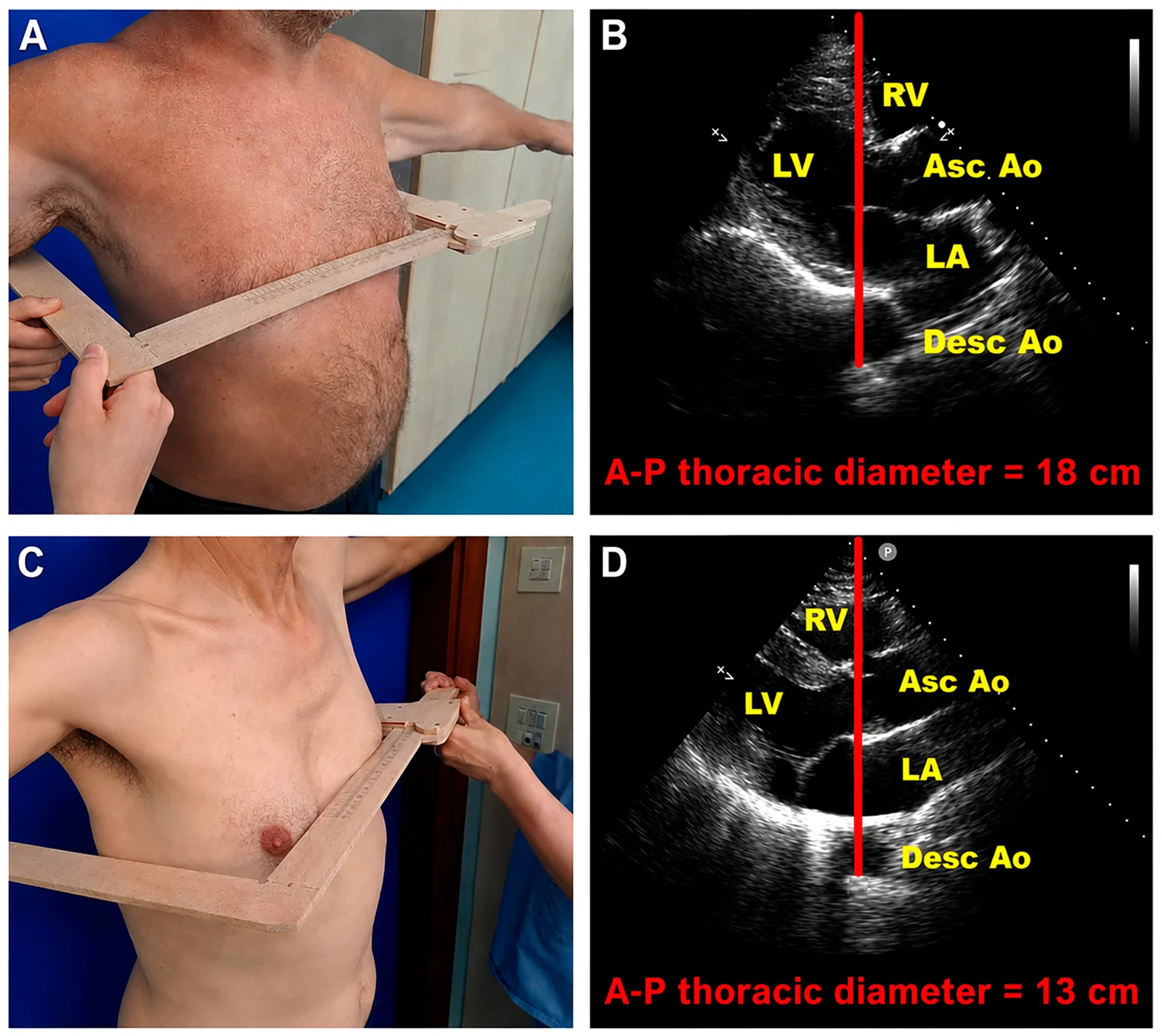
Assessment of Thoracic Geometry in Different Chest Configurations. Representative examples illustrating thoracic morphometric assessment in individuals with different chest configurations. (**A**) Measurement of the latero-lateral thoracic diameter using a centimeter-calibrated sliding-arm caliper in an individual with a wider, more circular thoracic configuration. (**B**) Echocardiographic measurement of the A-P thoracic diameter, defined as the distance between the sternum and the anterior surface of the vertebral body, using the descending aorta as an anatomical landmark; the red line indicates the measured A-P thoracic diameter, which is 18 cm in this example. (**C**) Measurement of the latero-lateral thoracic diameter using the same technique in an individual with a narrower, more concave thoracic configuration. (**D**) Corresponding echocardiographic assessment showing a reduced A-P thoracic diameter of 13 cm, indicated by the red line. The MHI is calculated as the ratio of the latero-lateral to the A-P thoracic diameter. A-P, antero-posterior; Asc Ao, ascending aorta; Desc Ao, descending aorta; LA, left atrium; LV, left ventricle; MHI, modified Haller index; RV, right ventricle.

**Figure 7:**
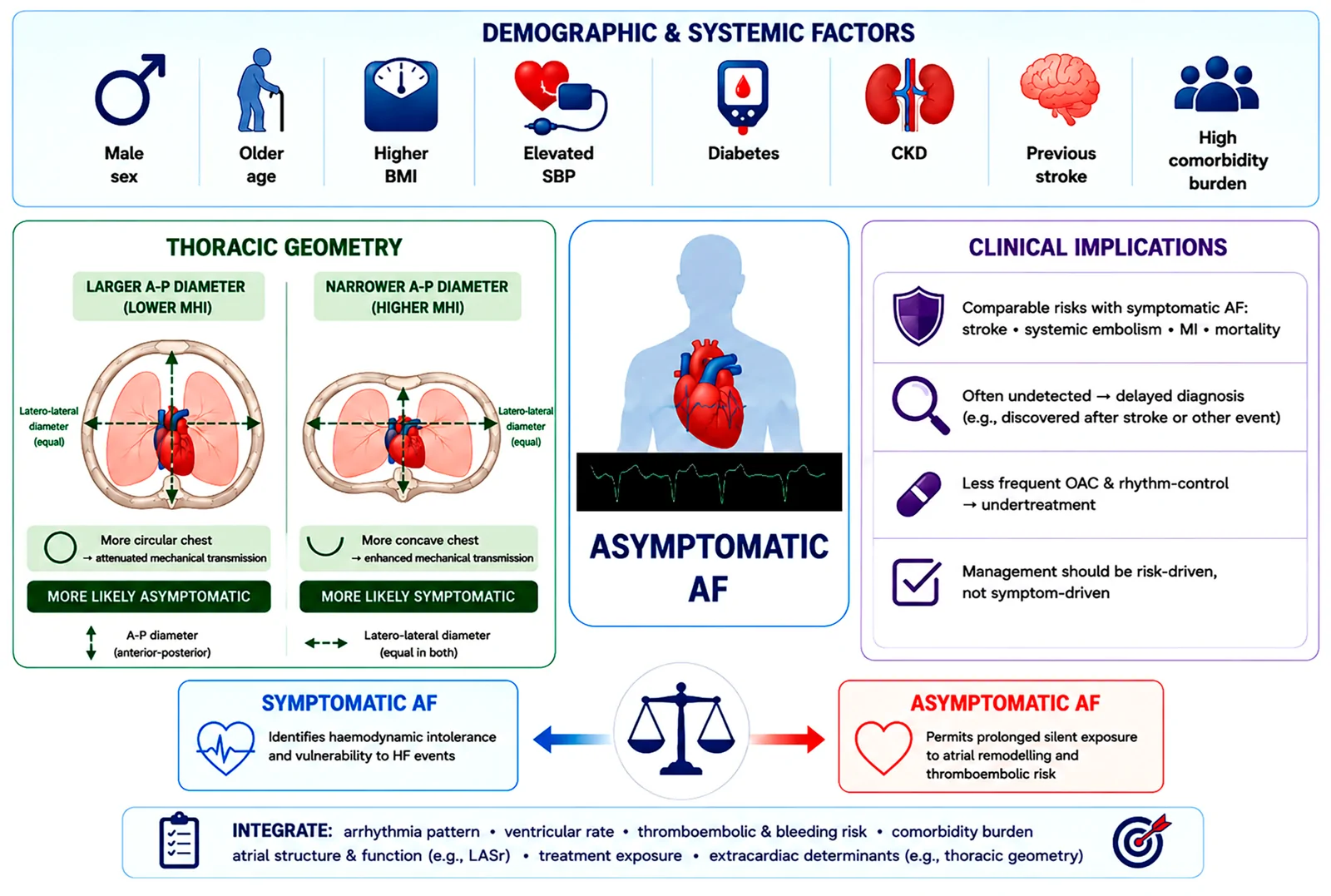
Schematic representation of the principal factors potentially associated with asymptomatic AF and their clinical implications. Demographic and systemic characteristics linked to reduced symptom perception include male sex, older age, higher body mass index, elevated systolic blood pressure, diabetes, chronic kidney disease, previous stroke, and greater comorbidity burden. Thoracic geometry may represent an additional extracardiac modulator of symptom perception: a larger antero-posterior thoracic diameter and lower modified Haller index may attenuate mechanical transmission of cardiac motion to the chest wall, whereas a narrower antero-posterior diameter may enhance symptom perception. Despite greater haemodynamic tolerance, asymptomatic AF should not be considered biologically benign, as thromboembolic and mortality risks may remain comparable with those of symptomatic AF. Delayed recognition and lower exposure to anticoagulation and rhythm-control strategies may further contribute to adverse outcomes. The proposed framework emphasizes risk-driven rather than symptom-driven AF assessment and management. A-P, antero-posterior; AF, atrial fibrillation; BMI, body mass index; CKD, chronic kidney disease; HF, heart failure; LA, left atrial; LASr, left atrial reservoir strain; MHI, modified Haller index; MI, myocardial infarction; OAC, oral anticoagulation; SBP, systolic blood pressure.

**Figure 8:**
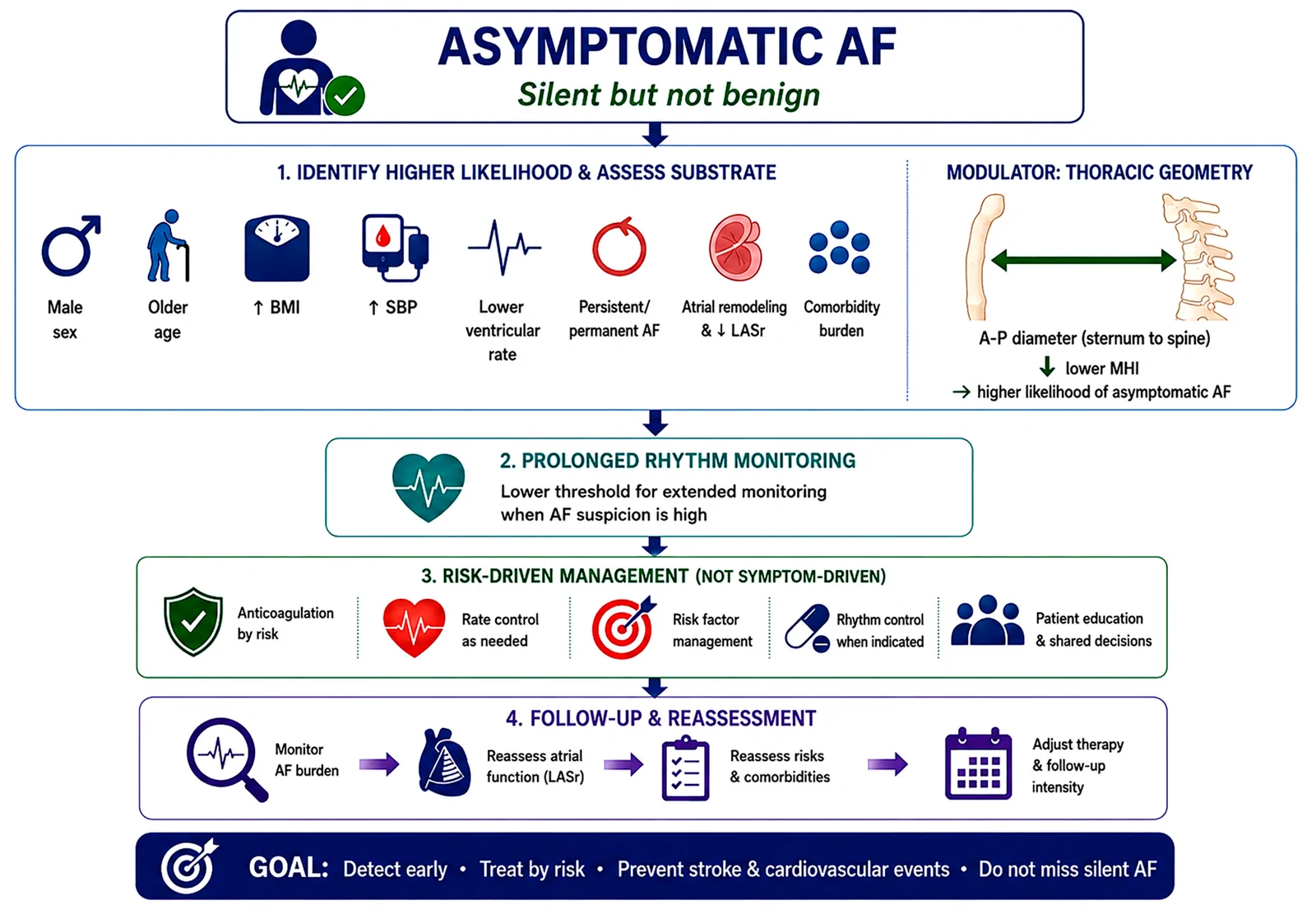
Proposed Clinical Management Framework for Asymptomatic Atrial Fibrillation. Recognition of a phenotype associated with silent AF should prompt comprehensive evaluation of arrhythmia pattern and ventricular response, atrial remodeling and mechanical function, comorbidity burden, and thromboembolic and bleeding risk, with prolonged rhythm monitoring when clinically appropriate. Thoracic geometry may represent an additional extracardiac modulator of symptom perception. Management should remain risk-driven rather than symptom-driven and include appropriate anticoagulation, rate or rhythm control when indicated, cardiovascular risk-factor modification, and longitudinal reassessment of AF burden, atrial function, comorbidities, and treatment. A-P, antero-posterior; AF, atrial fibrillation; BMI, body mass index; LASr, left atrial reservoir strain; MHI, modified Haller index; SBP, systolic blood pressure.

**Table 1:** Characteristics of the Studies Included in the Systematic Review [24,27,35,36,37,38,39,40,41,42,43,44,45,46,47,48,49,50,51,52,53,54,55].

Study, Year, Country	Study Design	Population	Asymptomatic AF/Comparator Group, n
Wong J.S., 2013, Malaysia [35]	Single-center retrospective audit	Consecutive hypertensive-clinic patients aged ≥ 30 years without typical AF symptoms; known AF and previous thromboembolic events excluded; asymptomatic AF detected by single 12-lead ECG	15/no symptomatic-AF comparator
Potpara T.S., 2013, Serbia [36]	Prospective, single-center observational registry	Patients with first-diagnosed nonvalvular AF	146/954
Rienstra M., 2014, The Netherlands [37]	Post hoc analysis of randomized controlled trial (RACE)	Patients with recurrent persistent AF enrolled in RACE	157/365
Stamboul K., 2014, France [38]	Prospective, single-center observational study	Patients with acute MI undergoing continuous ECG monitoring; silent/asymptomatic AF defined as episodes ≥ 30 s	135/45
Park H.C., 2015, South Korea [39]	Prospective population-/community-based cohort	Relatively healthy community-dwelling adults aged ≥ 21 years from Yangpyeong County; AF detected by resting 12-lead ECG	54/no symptomatic-AF comparator
Witkowski M., 2017, Poland [40]	Prospective, single-center observational study	Dual-chamber pacemaker recipients with paroxysmal AF, classified according to EHRA symptom status	28/22
Guerra F., 2017, Europe (35 ESC countries) [41]	Prospective, multicenter observational registry (Euro Heart Survey)	AF patients with symptom status recorded at baseline	1155/2452
Esato M., 2017, Japan [42]	Prospective, community-based observational registry	Community-based AF cohort stratified by AF type and symptom status	1971/1778
Wong J.A., 2018, multinational [43]	Prospective cohort; ASSERT analysis	Pacemaker/ICD recipients without previous clinical AF and with asymptomatic SCAF > 6 min to ≤24 h	415/no symptomatic-AF comparator
Thind M., 2018, USA [44]	Prospective, multicenter nationwide observational registry (ORBIT-AF)	Patients with nonvalvular AF classified as EHRA I vs. EHRA II–IV	3582/5737
Bertelsen L., 2020, Denmark [45]	Prospective cohort; CMR substudy of LOOP with continuous ILR monitoring	Participants undergoing continuous ILR monitoring with incident AF detected during follow-up	79/no symptomatic-AF comparator
Gibbs H., 2021, multinational (35 countries) [46]	Prospective, multicenter observational registry (GARFIELD-AF)	Patients with newly diagnosed AF, classified according to presence or absence of AF-related symptoms	13,235/38,797
Wang S., 2022, China [47]	Single-center prospective cohort	CIED recipients without previous AF/AFL/AT; SCAF defined as device-detected atrial tachyarrhythmia > 6 min confirmed by intracardiac electrograms	225 SCAF/999 non-SCAF
Xie P.X., 2022, 6 Gulf countries [48]	Multinational, multicenter prospective observational registry (Gulf-SAFE)	Patients with AF classified according to symptom status	541/1502
Taş U., 2022, Turkey [49]	Prospective, single-center case–control study	Adults with untreated or irregularly treated essential hypertension undergoing simultaneous 24-h ECG and ABPM monitoring	32 asymptomatic PAF/comparator group
Lin J., 2023, China [50]	Cross-sectional, multicenter national registry (CCC-AF)	Hospitalized patients with nonvalvular AF classified as EHRA I vs. EHRA II–IV	4505/48,025
Boriani G., 2024, Europe [27]	Prospective, multicenter observational registry	AF patients stratified by EHRA symptom status, including patients with and without HF and LVEF > 40% or ≤40%	3736/4360
Sonaglioni A., 2024, Italy [24]	Retrospective, single-center observational study	Patients with persistent AF scheduled for early electrical cardioversion, evaluated according to symptom status	25/90
Lee S.J., 2024, South Korea [51]	Prospective, multicenter observational registry (CODE-AF)	Patients with nonvalvular AF classified according to symptom status	5883/4327
Ahluwalia N., 2024, UK/USA [52]	Retrospective observational database study	Patients with ICM-detected AF, classified according to absence or presence of symptomatic AF episodes	9491/1776
Minguito-Carazo C., 2026, Spain [53]	Prospective, multicenter observational registry	AF patients classified as EHRA I vs. EHRA IIa–IV	336/661
Mao J., 2026, USA [54]	Prospective cohort study (VITAL Rhythm)	Participants with newly diagnosed AF classified as asymptomatic or symptomatic	365/746
Peng X., 2026, China [55]	Prospective, multicenter, hospital-based observational registry (China-AF)	Patients with AF classified as EHRA I vs. EHRA ≥ II	1345/14,258

Unless otherwise specified, the comparator group consisted of patients with symptomatic AF. ABPM, ambulatory blood pressure monitoring; AF, atrial fibrillation; AFL, atrial flutter; AT, atrial tachycardia; CCC-AF, Improving Care for Cardiovascular Disease in China–Atrial Fibrillation; CIED, cardiac implantable electronic device; CMR, cardiovascular magnetic resonance; CODE-AF, Comparison Study of Drugs for Symptom Control and Complication Prevention of Atrial Fibrillation; ECG, electrocardiography; EHRA, European Heart Rhythm Association; ESC, European Society of Cardiology; GARFIELD-AF, Global Anticoagulant Registry in the FIELD–Atrial Fibrillation; Gulf-SAFE, Gulf Survey of Atrial Fibrillation Events; HF, heart failure; ICD, implantable cardioverter-defibrillator; ICM, insertable cardiac monitor; ILR, implantable loop recorder; LVEF, left ventricular ejection fraction; MI, myocardial infarction; ORBIT-AF, Outcomes Registry for Better Informed Treatment of Atrial Fibrillation; PAF, paroxysmal atrial fibrillation; RACE, Rate Control versus Electrical Cardioversion for Persistent Atrial Fibrillation; SCAF, subclinical atrial fibrillation.

**Table 2:** Demographic and Clinical Characteristics and Atrial Fibrillation Phenotype According to Symptom Status.

Parameter	Asymptomatic AF	Studies/Total N	Symptomatic AF	Studies/Total N
Age, years	70.88 (53.1–80.0)	23/47,456	68.60 (52.6–81.0)	18/126,894
Male sex, %	63.21%	23/47,456	55.39%	18/126,894
BMI, kg/m^2^	26.39 (23.0–29.1)	13/31,373	26.74 (23.1–29.3)	9/71,550
L-L thoracic diameter, cm	34.7 (34.7–34.7)	1/25	33.8 (33.8–33.8)	1/90
A-P thoracic diameter, cm	17.4 (17.4–17.4)	1/25	14.3 (14.3–14.3)	1/90
MHI	2.00 (2.00–2.00)	1/25	2.40 (2.40–2.40)	1/90
Hypertension, %	70.59%	23/47,456	65.53%	18/126,894
Smoking, %	31.86%	12/17,028	22.82%	10/76,390
Alcohol consumption, %	22.25%	8/13,254	14.74%	7/70,518
Diabetes, %	24.80%	22/47,441	20.48%	18/126,894
Dyslipidemia, %	46.64%	9/22,989	46.71%	8/65,450
CAD, %	23.76%	21/47,076	18.89%	17/126,148
Stroke/TIA, %	18.23%	18/41,832	10.66%	14/106,576
PAD, %	12.82%	8/38,000	11.54%	7/111,413
Heart failure, %	17.35%	13/35,724	19.41%	10/101,772
CKD, %	14.48%	13/45,947	8.46%	12/123,718
COPD, %	6.89%	11/12,203	4.41%	10/73,666
Thyroid disease, %	5.91%	5/3434	4.67%	5/18,980
Hiatus hernia, %	1.40%	1/146	3.70%	1/954
Dementia, %	0.89%	1/336	0.61%	1/661
Anemia, %	14.19%	3/5263	10.42%	3/20,656
Major bleeding, %	3.06%	3/7017	1.57%	3/51,305
Any bleeding event, %	3.52%	3/17,307	2.65%	3/43,818
Malignancy, %	9.28%	2/6219	8.90%	2/4988
Obesity, %	40.50%	1/336	41.10%	1/661
Pain/discomfort, %	25.60%	1/336	33.20%	1/661
Anxiety/depression, %	23.70%	1/336	33.40%	1/661
CCI	6.8 (6.8–6.8)	1/25	2.3 (2.3–2.3)	1/90
First-diagnosed AF, %	48.31%	10/33,370	48.91%	9/106,607
Paroxysmal AF, %	34.90%	10/32,836	37.37%	10/106,266
Persistent AF, %	23.86%	9/32,808	21.70%	9/106,244
Long-standing persistent/permanent AF, %	23.30%	10/33,144	14.85%	9/106,815
Heart rate, bpm	78.64 (68.7–114.0)	14/31,370	86.46 (70.0–141.0)	11/102,592
SBP, mmHg	128.82 (122.7–148.3)	9/20,325	130.67 (123.2–144.0)	6/93,512
DBP, mmHg	78.49 (74.0–85.0)	9/20,325	79.19 (74.0–85.0)	6/93,512

Continuous variables are summarized as sample-size-weighted estimates derived from individual studies, with the corresponding range of study-level central values reported in parentheses. Categorical variables are expressed as overall proportions calculated from the available aggregated data. The number of contributing investigations and participants differed across individual parameters, and estimates for asymptomatic and symptomatic AF were not consistently obtained from the same sets of studies. Therefore, the reported values represent variable-specific descriptive summaries of heterogeneous datasets rather than directly comparable estimates between symptom-status groups. No inferential between-group analyses or statistical significance testing were performed on aggregated data. Accordingly, observed numerical differences should not be interpreted as pooled comparative effects, statistically established associations, or differences independently attributable to AF symptom status. A-P, antero-posterior; AF, atrial fibrillation; BMI, body mass index; CAD, coronary artery disease; CCI, Charlson Comorbidity Index; CKD, chronic kidney disease; COPD, chronic obstructive pulmonary disease; DBP, diastolic blood pressure; L-L, latero-lateral; MHI, modified Haller index; PAD, peripheral artery disease; SBP, systolic blood pressure; TIA, transient ischemic attack.

**Table 3:** Laboratory, Electrocardiographic, and Echocardiographic Characteristics According to Atrial Fibrillation Symptom Status.

Parameter	Asymptomatic AF	Studies/Total N	Symptomatic AF	Studies/Total N
Hb, g/dL	14.05 (13.1–14.3)	3/7260	14.21 (13.9–14.3)	2/18,585
Creatinine, mg/dL	0.94 (0.86–1.35)	4/6510	0.97 (0.90–1.18)	2/5829
eGFR, mL/min/1.73 m^2^	85.20 (51.0–89.7)	9/12,745	88.29 (49.3–90.9)	7/69,246
Total cholesterol, mg/dL	158.00 (154.7–195.9)	4/5936	161.34 (158.5–170.9)	2/62,283
HDL-C, mg/dL	46.05 (44.2–51.3)	3/1431	46.00 (46.0–46.0)	1/14,258
Triglycerides, mg/dL	129.50 (125.2–137.1)	3/1431	132.90 (132.9–132.9)	1/14,258
LDL-C, mg/dL	94.42 (92.8–129.0)	4/6028	91.92 (88.9–102.1)	2/62,283
Glucose, mg/dL	108.74 (106.3–131.3)	4/1566	108.18 (108.1–132.2)	2/14,303
Homocysteine, μmol/L	15.3 (15.3–15.3)	1/225	13.1 (13.1–13.1)	1/999
Uric acid, mg/dL	6.23 (5.50–6.41)	2/279	5.57 (5.57–5.57)	1/999
ALT, U/L	21 (21–21)	1/54	—	0
NT-proBNP, pg/mL	551.29 (550–855.1)	2/5908	446.48 (277.2–450)	2/4417
Troponin I, ng/mL	0.030 (0.030–0.030)	1/5883	0.015 (0.015–0.015)	1/4327
hs-CRP, mg/dL	0.40 (0.40–0.40)	1/5883	0.70 (0.70–0.70)	1/4327
QRS, ms	96.0 (96–96)	1/5883	94.0 (94–94)	1/4327
LVH on ECG, %	16.57%	3/1010	24.40%	1/1502
IVS, mm	9.86 (9.8–11.8)	4/1588	9.71 (9.7–10.0)	2/14,623
PW, mm	9.57 (9.0–11.6)	4/1588	9.32 (9.3–10.0)	2/14,623
LVEDD, mm	49.42 (49.0–51.0)	4/1588	48.42 (48.3–53.0)	2/14,623
LVMi, g/m^2^	94.60 (64.8–114.4)	4/1481	91.37 (86.9–91.4)	2/14,348
Peak E, cm/s	92.30 (85.9–103.1)	2/86	—	0
E/e′	10.46 (10.4–17.7)	3/5940	10.01 (10.0–10.7)	2/4417
LA A-P diameter, mm	43.28 (38.0–47.1)	13/16,177	41.53 (37.0–45.5)	11/74,727
LAVi, mL/m^2^	41.79 (29.5–48.4)	4/6122	41.01 (33.9–41.2)	3/4462
LASr, %	25.56 (18.5–27.8)	2/104	28.20 (28.2–28.2)	1/90
LAA global peak strain, %	7.0 (7.0–7.0)	1/25	11.7 (11.7–11.7)	1/90
LVEF, %	58.49 (46.8–66.2)	17/20,611	57.97 (40.0–62.0)	13/79,473
Mitral regurgitation, %	10.22%	2/361	16.51%	2/751
Aortic regurgitation, %	1.80%	1/336	3.00%	1/661
Aortic stenosis, %	3.27%	1/336	5.10%	1/661
RVIT, mm	35.0 (35.0–35.0)	1/25	28.7 (28.7–28.7)	1/90
TAPSE, mm	15.4 (15.4–15.4)	1/25	21.5 (21.5–21.5)	1/90
sPAP, mmHg	48.5 (48.5–48.5)	1/25	31.3 (31.3–31.3)	1/90

Continuous variables are presented as sample-size-weighted study-level estimates, with the range of study-level central estimates in parentheses. Categorical variables are presented as aggregated proportions. The number of contributing investigations and participants differed across individual parameters, and estimates for asymptomatic and symptomatic AF were not consistently obtained from overlapping study populations. Therefore, the reported values should be regarded as variable-specific descriptive summaries of heterogeneous datasets rather than directly comparable estimates between symptom-status groups. No inferential statistical comparisons were undertaken using aggregated data. Accordingly, observed numerical variations should not be interpreted as pooled comparative effects, statistically established differences, or independent associations attributable to AF symptom status. AF, atrial fibrillation; ALT, alanine aminotransferase; A-P, antero-posterior; ECG, electrocardiogram; eGFR, estimated glomerular filtration rate; Hb, hemoglobin; HDL-C, high-density lipoprotein cholesterol; hs-CRP, high-sensitivity C-reactive protein; IVS, interventricular septum; LA, left atrial; LAA, left atrial appendage; LASr, left atrial reservoir strain; LAVi, left atrial volume index; LDL-C, low-density lipoprotein cholesterol; LVEDD, left ventricular end-diastolic diameter; LVEF, left ventricular ejection fraction; LVH, left ventricular hypertrophy; LVMi, left ventricular mass index; NT-proBNP, N-terminal pro-B-type natriuretic peptide; PW, posterior wall; RVIT, right ventricular inflow tract; sPAP, systolic pulmonary artery pressure; TAPSE, tricuspid annular plane systolic excursion.

**Table 4:** Risk Scores, Cardiac Devices, and Therapeutic Management According to Atrial Fibrillation Symptom Status.

Parameter	Asymptomatic AF	Studies/Total N	Symptomatic AF	Studies/Total N
CHA_2_DS_2_-VASc score	3.05 (1.0–5.8)	18/37,729	2.93 (1.2–4.0)	16/125,073
HAS-BLED score	1.17 (0.74–4.60)	6/19,987	1.11 (0.69–2.80)	6/103,333
Permanent PMK, %	17.35%	6/11,288	21.25%	5/13,537
ICD, %	1.28%	4/7678	1.47%	3/7778
Antiplatelets, %	21.69%	16/37,006	17.83%	14/123,346
Anticoagulants, %	58.85%	14/44,848	46.82%	14/124,123
ACE-i/ARBs, %	48.42%	11/23,033	36.95%	9/29,741
ARNI, %	0.30%	1/9491	0.30%	1/1776
Beta-blockers, %	51.59%	12/23,795	51.53%	10/17,013
Digoxin, %	13.29%	7/13,449	15.17%	7/14,486
Anti-arrhythmic drugs, %	20.16%	9/26,811	17.12%	8/76,197
CCB, %	30.37%	5/8186	29.27%	4/7126
Diuretics, %	33.63%	10/23,352	34.44%	9/16,991
Spironolactone/MRA, %	9.15%	2/13,227	14.73%	2/6136
Statins, %	37.72%	8/8541	36.47%	6/21,198
Rhythm control, %	39.63%	6/9181	56.88%	6/25,471
Catheter ablation, %	12.02%	6/15,195	15.31%	6/71,501
Electrical cardioversion, %	9.78%	5/6049	4.12%	5/51,250

Continuous variables are presented as sample-size-weighted study-level estimates, with the range of study-level central estimates in parentheses. Categorical variables are presented as aggregated proportions. The number of contributing studies and participants varied across individual parameters, and estimates for asymptomatic and symptomatic AF were not necessarily derived from identical sets of studies. The reported values therefore represent variable-specific descriptive summaries of heterogeneous datasets rather than matched or directly comparable patient populations. Accordingly, these findings are presented exclusively as descriptive summaries, without inferential between-group statistical testing. Numerical differences should not be interpreted as pooled comparative effects or evidence of independent associations with AF symptom status and should not be attributed to AF symptom status alone. ACE-i, angiotensin-converting enzyme inhibitor; AF, atrial fibrillation; ARB, angiotensin receptor blocker; ARNI, angiotensin receptor–neprilysin inhibitor; CCB, calcium-channel blocker; CHA_2_DS_2_-VASc, congestive heart failure, hypertension, age ≥ 75 years, diabetes mellitus, stroke/transient ischemic attack/thromboembolism, vascular disease, age 65–74 years, and sex category; HAS-BLED, hypertension, abnormal renal/liver function, stroke, bleeding history or predisposition, labile international normalized ratio, elderly, and drugs/alcohol; ICD, implantable cardioverter-defibrillator; MRA, mineralocorticoid receptor antagonist; PMK, pacemaker.

**Table 5:** Characteristics and Principal Findings of Studies Investigating Device-Detected Subclinical and Continuously Monitored Silent Atrial Fibrillation [38,40,43,45,47,49,52].

Study	Detection Method	Study Population/Comparator	Principal Findings
Stamboul K. [38]	Continuous ECG monitoring	135 silent AF/45 symptomatic AF/669 no AF	Higher in-hospital HF and mortality in silent AF vs. no AF.
Witkowski M. [40]	Dual-chamber pacemaker	28 asymptomatic/22 symptomatic AF	Lower ventricular rate and shorter episodes in asymptomatic AF.
Wong J.A. [43]	Pacemaker/ICD (ASSERT)	415 SCAF; progression vs. no progression	SCAF progression predicted HF hospitalization (HR 4.58).
Bertelsen L. [45]	Implantable loop recorder (LOOP)	79 incident AF/124 no AF	LA enlargement and impaired atrial function predicted incident AF.
Wang S. [47]	Cardiac implantable electronic devices	225 SCAF/999 no SCAF	Homocysteine and uric acid independently predicted SCAF.
Taş U. [49]	24-h Holter ECG	32 asymptomatic PAF/232 no PAF	Morning BP surge and LA diameter independently predicted PAF.
Ahluwalia N. [52]	Insertable cardiac monitor	9491 without symptomatic episodes/1776 with symptomatic episodes	Higher ventricular rate, episode duration, and rhythm irregularity in symptomatic AF.

Studies are presented according to the modality of AF detection, including implantable cardiac devices and continuous external ECG monitoring. Comparator populations and clinical endpoints varied across investigations. Findings represent study-specific results without statistical pooling or additional inferential comparisons. Device-detected subclinical AF and clinically documented asymptomatic AF were considered distinct clinical entities. AF, atrial fibrillation; BP, blood pressure; ECG, electrocardiogram; HF, heart failure; HR, hazard ratio; ICD, implantable cardioverter-defibrillator; LA, left atrial; PAF, paroxysmal atrial fibrillation; SCAF, subclinical atrial fibrillation.

**Table 6:** Design-Specific Methodological Quality and Risk-of-Bias Assessment of the 23 Included Studies [24,27,35,36,37,38,39,40,41,42,43,44,45,46,47,48,49,50,51,52,53,54,55] Using Joanna Briggs Institute (JBI) Critical Appraisal Criteria.

Study	Study Design	JBI Appraisal Tool	Main Methodological Concerns
Wong J.S. [35]	Retrospective audit	Analytical cross-sectional	Single ECG assessment; incomplete clinical records
Potpara T.S. [36]	Prospective registry	Cohort	Residual confounding; symptom classification
Rienstra M. [37]	Post hoc RACE analysis	Cohort	Post hoc symptom comparison; potential confounding
Stamboul K. [38]	Prospective observational	Cohort	Acute MI setting; residual confounding
Park H.C. [39]	Community-based study	Analytical cross-sectional	Single ECG assessment; potential underdetection of AF
Witkowski M. [40]	Prospective observational	Cohort	Small sample; device-selected population
Guerra F. [41]	Prospective registry	Cohort	Symptom assessment; residual confounding
Esato M. [42]	Prospective registry	Cohort	Detection bias; treatment-related confounding
Wong J.A. [43]	ASSERT secondary analysis	Cohort	Device-selected population; progression-related confounding
Thind M. [44]	Prospective registry	Cohort	Symptom classification; residual confounding
Bertelsen L. [45]	LOOP observational substudy	Cohort	Selected CMR population; limited events
Gibbs H. [46]	Prospective registry	Cohort	Baseline symptom classification; residual confounding
Wang S. [47]	Prospective cohort	Cohort	Device selection; biomarker-related confounding
Xie P.X. [48]	Prospective registry	Cohort	Emergency-care selection; residual confounding
Taş U. [49]	Case–control	Case–control	Participant selection; potential confounding
Lin J. [50]	Cross-sectional registry	Analytical cross-sectional	Hospital-based selection; absence of temporal inference
Boriani G. [27]	Prospective registry	Cohort	HF subgroup confounding; symptom classification
Sonaglioni A. [24]	Retrospective observational	Case–control	Small sample; selection and residual confounding
Lee S.J. [51]	Prospective registry	Cohort	Symptom classification; residual confounding
Ahluwalia N. [52]	Retrospective database study	Cohort	Symptom underreporting; device-selected population
Minguito-Carazo C. [53]	Prospective registry	Cohort	Symptom classification; residual confounding
Mao J. [54]	Prospective cohort	Cohort	Symptom ascertainment; treatment-related confounding
Peng X. [55]	Prospective registry	Cohort	Hospital-based selection; residual confounding

Design-specific methodological appraisal of the 23 studies included in the systematic review, structured according to the Joanna Briggs Institute (JBI) critical appraisal framework. Studies were categorized according to their actual analytical design, and the corresponding JBI appraisal instruments for cohort, analytical cross-sectional, or case–control studies were selected. Secondary observational analyses derived from randomized clinical trials were assessed according to their observational research questions rather than the randomized design of the parent trials. The table summarizes the principal methodological concerns relevant to the interpretation of study-specific findings, including participant selection, exposure and outcome ascertainment, potential confounding, symptom classification, and follow-up completeness. Numerical quality scores and arbitrary score-based classifications were not used. AF, atrial fibrillation; ASSERT, Asymptomatic Atrial Fibrillation and Stroke Evaluation in Pacemaker Patients and the Atrial Fibrillation Reduction Atrial Pacing Trial; CMR, cardiovascular magnetic resonance; ECG, electrocardiogram; HF, heart failure; JBI, Joanna Briggs Institute; LOOP, Atrial Fibrillation Detected by Continuous ECG Monitoring Using Implantable Loop Recorder to Prevent Stroke in High-Risk Individuals; MI, myocardial infarction; RACE, Rate Control versus Electrical Cardioversion for Persistent Atrial Fibrillation.

## Data Availability

The dataset generated from the extraction of data from the included studies will be made openly accessible through Zenodo (https://zenodo.org; accessed 7 August 2026).

## Supplementary Materials

The following supporting information can be downloaded at: https://www.mdpi.com/article/10.3390/jcm15208191/s1, File S1: PRISMA 2020 checklist; File S2: The full INPLASY record (https://inplasy.com/inplasy-2026-9-0012/, accessed on 3 September 2026); File S3: Database-specific literature search strategies for PubMed/MEDLINE, Scopus, and EMBASE, including search terms, Boolean operators, and supplementary information on record management and manual searching; File S4: Bibliographic Details of Reports Sought for Full-Text Retrieval but Not Obtained; File S5: Domain-level methodological appraisal of the 23 included studies [24,27,35,36,37,38,39,40,41,42,43,44,45,46,47,48,49,50,51,52,53,54,55] using design-specific Joanna Briggs Institute (JBI) critical appraisal checklists.
