# Supplementary material for: The Silent Face of Atrial Fibrillation: A Systematic Review of Associated Factors, Clinical Phenotype, and Outcomes
Source: J Clin Med. 2026 Oct 21;15(20):8191. doi: 10.3390/jcm15208191 (PMC13706439; doi:10.3390/jcm15208191)
Supplement: Supplementary file 1 [file jcm-15-08191-s001.zip › Supplementary Material File S3.pdf]

Supplementary Material File S3

Database-specific search strategies and search-record documentation

Scope and provenance

The literature searches covered PubMed/MEDLINE, Scopus, and EMBASE from database inception through September 1, 2026. As the original contemporaneous search histories were unavailable, the database-specific search strategies were reconstructed using the predefined search concepts and adapted to the indexing systems and syntax of each database. These strategies were subsequently executed in the corresponding databases, and the resulting retrieval counts were incorporated into the revised manuscript and PRISMA 2020 flow diagram. The reconstructed strategies are distinguished from the original historical searches to ensure transparency regarding the literature identification process.

1. PubMed/MEDLINE

Reconstructed query (PubMed field tags; MeSH and title/abstract terms):

("Atrial Fibrillation"[Mesh] OR "atrial fibrillation"[tiab] OR "AF"[tiab]) AND ("asymptomatic"[tiab] OR "silent"[tiab] OR "subclinical"[tiab] OR "symptomatic"[tiab] OR "symptom status"[tiab] OR "AF symptoms"[tiab] OR "device-detected"[tiab] OR "atrial high-rate episode"[tiab] OR "atrial high-rate episodes"[tiab]) AND ("risk factors"[MeSH Terms] OR "prognosis"[MeSH Terms] OR "mortality"[MeSH Terms] OR "stroke"[MeSH Terms] OR "heart failure"[MeSH Terms] OR predictor*[tiab] OR determinant*[tiab] OR "risk factor"[tiab] OR "risk factors"[tiab] OR "clinical characteristics"[tiab] OR "atrial remodeling"[tiab] OR outcome*[tiab] OR prognos*[tiab] OR mortalit*[tiab] OR stroke[tiab] OR "systemic embolism"[tiab] OR "heart failure"[tiab] OR hospitali*[tiab] OR bleeding[tiab] OR progression[tiab] OR biomarker*[tiab] OR echocardiograph*[tiab])

Coverage: database inception to 1 September 2026; no language or geographic restriction. The MeSH elements are proposed for reproducibility and are not independently verified as having been used in the original search.

2. Scopus

Reconstructed advanced-search query (TITLE-ABS-KEY):

TITLE-ABS-KEY("atrial fibrillation" OR "AF") AND TITLE-ABS-KEY(asymptomatic OR silent OR subclinical OR symptomatic OR "symptom status" OR "AF symptoms" OR "device-detected" OR "atrial high-rate episode" OR "atrial high-rate episodes") AND TITLE-ABS-KEY("risk factors" OR predictor* OR determinant* OR "clinical characteristics" OR "atrial remodeling" OR outcome* OR prognos* OR mortalit* OR stroke OR "systemic embolism" OR "heart failure" OR hospitali* OR bleeding OR progression OR biomarker* OR echocardiograph*)

Coverage: database inception to 1 September 2026; no language or geographic restriction. Scopus TITLE-ABS-KEY uses indexed keywords and text fields, not MeSH or Emtree syntax.

3. EMBASE

Reconstructed query for the Embase.com interface (Emtree and title/abstract terms):

('atrial fibrillation'/exp OR 'atrial fibrillation':ti,ab OR 'AF':ti,ab) AND ('asymptomatic':ti,ab OR 'silent':ti,ab OR 'subclinical':ti,ab OR 'symptomatic':ti,ab OR 'symptom status':ti,ab OR 'AF symptoms':ti,ab OR 'device-detected':ti,ab OR 'atrial high-rate episode':ti,ab OR 'atrial high-rate episodes':ti,ab) AND ('risk factor'/exp OR 'prognosis'/exp OR 'mortality'/exp OR 'stroke'/exp OR 'heart failure'/exp OR 'predictor*':ti,ab OR 'determinant*':ti,ab OR 'clinical characteristics':ti,ab OR 'atrial remodeling':ti,ab OR 'outcome*':ti,ab OR 'prognos*':ti,ab OR 'mortalit*':ti,ab OR 'systemic embolism':ti,ab OR 'hospitali*':ti,ab OR 'bleeding':ti,ab OR 'progression':ti,ab OR 'biomarker*':ti,ab OR 'echocardiograph*':ti,ab)

Coverage: database inception to 1 September 2026; no language or geographic restriction. The historical EMBASE interface is not documented; the syntax above is for Embase.com and should not be assumed to reproduce an Ovid search.

4. Record handling and deduplication

The database searches identified 41,412 records, comprising 8,173 from PubMed/MEDLINE, 12,865 from Scopus, and 20,374 from EMBASE. Bibliographic records were examined for duplication using automated matching procedures based on digital object identifiers (DOIs), PubMed identifiers (PMIDs), and normalized article titles. Based on the duplicate proportion identified in the available bibliographic exports, the total number of duplicate records was estimated at 17,017, leaving approximately 24,395 records after deduplication, as illustrated in the PRISMA 2020 flow diagram (Figure 1). The subsequent study selection process included title and abstract screening, followed by full-text eligibility assessment according to predefined inclusion and exclusion criteria.

5. Supplementary manual searching

The reference lists of potentially eligible articles and included studies were additionally examined to identify relevant original publications not retrieved through the electronic database searches.

Interpretation

This supplement documents the search concepts, database-specific strategies, and adapted search syntax applied to PubMed/MEDLINE, Scopus, and EMBASE. The reconstructed search strategies were subsequently executed, identifying 41,412 records across the three databases, consistent with the revised manuscript and PRISMA 2020 flow diagram. The record-handling procedures, including the estimation of duplicate citations from the available bibliographic exports, are described to support methodological transparency and reproducibility. The original contemporaneous search histories were unavailable, and the reconstructed strategies should therefore be distinguished from the historical searches.
