# Supplementary material for: The Silent Face of Atrial Fibrillation: A Systematic Review of Associated Factors, Clinical Phenotype, and Outcomes
Source: J Clin Med. 2026 Oct 21;15(20):8191. doi: 10.3390/jcm15208191 (PMC13706439; doi:10.3390/jcm15208191)
Supplement: Supplementary file 1 [file jcm-15-08191-s001.zip › Supplementary Material File S4.pdf]

1. Gunawardene M, Hartmann J, Willems S. Asymptomatisches Vorhofflimmern : Screening und Therapie [Asymptomatic atrial fibrillation : Screening and therapy]. Herzschrittmacherther Elektrophysiol. 2023 Jun;34(2):122-130. German. doi: 10.1007/s00399-023-00933-8. Epub 2023 Mar 13. PMID: 36912974.
2. Patten M. Asymptomatisches Vorhofflimmern bei systematischem Screening mittels Tele-EKG - Bedeutung für die Antikoagulation bei paroxysmalem Vorhofflimmern [Asymptomatic atrial fibrillation with systematic screening using tele-ECG--relevance for anticoagulation in paroxysmal atrial fibrillation]. Herzschrittmacherther Elektrophysiol. 2005 Sep;16(3):159-64. German. doi: 10.1007/s00399-005-0475-8. PMID: 16177942.
3. Okabe S, Sato A, Kasuya H, Sugiyama K, Endo S, Mitsunari H, Kamata K, Yamagata M, Katayanagi T. [Easy screening of outpatients for atrial fibrillation by analyzing fingertip pulse wave variation]. Nihon Ronen Igakkai Zasshi. 2018;55(3):402-410. Japanese. doi: 10.3143/geriatrics.55.402. PMID: 30122707.
4. Yang YH, Xu RQ, Zhang RF, Wei YS, Hong L, Sun J, Cong T, Xia YL. [Screening for asymptomatic atrial fibrillation in elder community populations in Dalian: a single center study]. Zhonghua Xin Xue Guan Bing Za Zhi. 2023 Oct 24;51(10):1056-1062. Chinese. doi: 10.3760/cma.j.cn112148-20230819-00097. PMID: 37859357.
5. Hao P, Ma CS, Dong JZ, Liu XP, Long DY, Fang DP, Hu FL, Yu RH, Tang RB, Lu CS, Liu XH. [Asymptomatic recurrence of atrial tachy-arrhythmia after circumferential pulmonary vein isolation in patients with atrial fibrillation]. Zhonghua Yi Xue Za Zhi. 2007 Jun 26;87(24):1669-72. Chinese. PMID: 17825144.
6. Aliot E. La fibrillation atriale asymptomatique [Silent atrial fibrillation]. Ann Cardiol Angeiol (Paris). 2009 Dec;58 Suppl 1:S22-4. French. doi: 10.1016/S0003-3928(09)73394-3. PMID: 20103174.
7. Brigadeau F, Lacroix D. Fibrillation atriale asymptomatique: que faire? [Asymptomatic atrial fibrillation: what to do?]. Rev Prat. 2013 Feb;63(2):196. French. PMID: 23513779.
8. Serafini A, Slawson D. Gli adulti con fibrillazione atriale sintomatica e asintomatica hanno una prognosi simile [Adults with symptomatic or asymptomatic atrial fibrillation have a similar prognosis]. Recenti Prog Med. 2025 Jul-Aug;116(7-8):458-459. Italian. doi: 10.1701/4530.45315. PMID: 40637528.
9. Wu L, Lu Y, Zheng L, Qiao YU, Chen G, Ding L, Hou B, Sun W, Liew R, Zhang S, Yao Y. Comparison of Radiofrequency Catheter Ablation Between Asymptomatic and Symptomatic Persistent Atrial Fibrillation: A Propensity Score Matched Analysis. J Cardiovasc Electrophysiol. 2016 May;27(5):531-5. doi: 10.1111/jce.12930. Epub 2016 Feb 12. PMID: 26773415.
10. Guenancia C, Garnier F, Mouhat B, Béjot Y, Maillot N, Fichot M, Fauchier L, Cottin Y. Dépistage et implications cliniques de la fibrillation atriale silencieuse [Screening and clinical implications of silent atrial fibrillation]. Rev Med Interne. 2018 Jul;39(7):574-579. French. doi: 10.1016/j.revmed.2017.08.006. Epub 2017 Sep 21. PMID: 28942937.
