# Supplementary material for: The Silent Face of Atrial Fibrillation: A Systematic Review of Associated Factors, Clinical Phenotype, and Outcomes
Source: J Clin Med. 2026 Oct 21;15(20):8191. doi: 10.3390/jcm15208191 (PMC13706439; doi:10.3390/jcm15208191)
Supplement: Supplementary file 1 [file jcm-15-08191-s001.zip › Supplementary Material File S5.pdf]

Supplementary File S5

Domain-level JBI critical appraisal of the 23 studies included in the systematic review

Each methodological criterion was assessed using the applicable JBI checklist. Y indicates Yes; N, No; U, Unclear; and NA, Not applicable. Judgments were based on information reported in the original publication.

A. Cohort and observational secondary analyses

Study	Ref.	Q1	Q2	Q3	Q4	Q5	Q6	Q7	Q8	Q9	Q10	Q11
Potpara T.S.	36	U	U	Y	Y	Y	U	Y	U	U	U	Y
Rienstra M.	37	U	U	Y	Y	Y	NA	Y	U	U	U	Y
Stamboul K.	38	U	U	Y	Y	Y	U	Y	U	U	U	Y
Witkowski M.	40	U	U	Y	Y	Y	U	Y	U	U	U	Y
Guerra F.	41	U	U	Y	Y	Y	U	Y	U	U	U	Y
Esato M.	42	U	U	Y	Y	Y	U	Y	U	U	U	Y
Wong J.A.	43	U	U	Y	Y	Y	U	Y	U	U	U	U
Thind M.	44	U	U	Y	Y	Y	U	Y	U	U	U	U
Bertelsen L.	45	U	U	Y	Y	Y	Y	Y	U	U	U	Y
Gibbs H.	46	U	U	Y	Y	Y	U	Y	U	U	U	Y
Wang S.	47	U	U	Y	Y	Y	U	Y	U	U	U	Y
Xie P.X.	48	U	U	Y	Y	Y	U	Y	U	U	U	Y
Boriani G.	27	U	U	Y	Y	Y	NA	Y	U	U	U	Y
Lee S.J.	51	U	U	Y	Y	Y	U	Y	U	U	U	Y
Ahluwalia N.	52	U	U	U	Y	Y	NA	U	U	U	U	Y
Minguito-Carazo C.	53	U	U	Y	Y	Y	U	Y	U	U	U	Y
Mao J.	54	U	U	Y	Y	Y	U	Y	U	U	U	Y
Peng X.	55	U	U	Y	Y	Y	U	Y	U	U	U	Y

Domain key: Q1: Groups similar and from same population | Q2: Exposure measured similarly in exposed/unexposed | Q3: Exposure measured validly and reliably | Q4: Confounding factors identified | Q5: Strategies to address confounding stated | Q6: Free of outcome at baseline | Q7: Outcomes measured validly and reliably | Q8: Follow-up period sufficient | Q9: Follow-up complete; losses explained | Q10: Strategies to address incomplete follow-up | Q11: Appropriate statistical analysis

B. Analytical cross-sectional studies

Study	Ref.	Q1	Q2	Q3	Q4	Q5	Q6	Q7	Q8
Wong J.S.	35	Y	U	Y	Y	U	U	U	Y
Park H.C.	39	U	U	Y	Y	Y	Y	U	Y
Lin J.	50	U	U	Y	Y	Y	Y	U	Y

Domain key: Q1: Inclusion criteria clearly defined | Q2: Study subjects and setting described | Q3: Exposure measured validly and reliably | Q4: Objective standard criteria for condition | Q5: Confounding factors identified | Q6: Strategies to deal with confounding stated | Q7: Outcomes measured validly and reliably | Q8: Appropriate statistical analysis

C. Case-control studies

Study	Ref.	Q1	Q2	Q3	Q4	Q5	Q6	Q7	Q8	Q9	Q10
Taş U.	49	U	U	U	Y	U	Y	Y	U	U	Y
Sonaglioni A.	24	U	U	U	Y	U	Y	Y	U	U	Y

Domain key: Q1: Groups comparable apart from condition | Q2: Cases and controls matched appropriately | Q3: Same criteria used to identify cases and controls | Q4: Exposure measured validly and reliably | Q5: Exposure measured identically in cases/controls | Q6: Confounding factors identified | Q7: Strategies to deal with confounding stated | Q8: Outcomes measured validly and reliably | Q9: Exposure period sufficient | Q10: Appropriate statistical analysis
