# Supplementary material for: The Silent Face of Atrial Fibrillation: A Systematic Review of Associated Factors, Clinical Phenotype, and Outcomes
Source: J Clin Med. 2026 Oct 21;15(20):8191. doi: 10.3390/jcm15208191 (PMC13706439; doi:10.3390/jcm15208191)
Supplement: Supplementary file 1 [file jcm-15-08191-s001.zip › Supplementary Material File S2.pdf]

INPLASY

The Silent Face of Atrial Fibrillation: A Systematic Review of Associated Factors, Clinical Phenotype, and Outcomes

INPLASY202690012

doi: 10.37766/inplasy2026.9.0012

Received: 3 September 2026

Published: 3 September 2026

Sonaglioni, A; Gramaglia, GF; Mondazzi, E; Nicolosi GL; Lombardo, M; Baravelli, M.

Corresponding author:
Andrea Sonaglioni

sonaglioniandrea@gmail.com

Author Affiliation:
IRCCS MultiMedica.

ADMINISTRATIVE INFORMATION

Support - Ministero della Salute Ricerca Corrente.

Review Stage at time of this submission - Completed but not published.

Conflicts of interest - None declared.

INPLASY registration number: INPLASY202690012

Amendments - This protocol was registered with the International Platform of Registered Systematic Review and Meta-Analysis Protocols (INPLASY) on 3 September 2026 and was last updated on 3 September 2026.

INTRODUCTION

Review question / Objective To systematically identify and characterize the demographic, clinical, anthropometric, laboratory, electrocardiographic, imaging, and therapeutic factors associated with asymptomatic atrial fibrillation (AF), and to evaluate the clinical outcomes of patients with asymptomatic AF in comparison with symptomatic AF or other relevant study-specific comparator groups.

Rationale Asymptomatic atrial fibrillation (AF) is increasingly identified through incidental findings, prolonged rhythm monitoring, and cardiac implantable electronic devices. However, the characteristics that distinguish patients with silent AF from those with symptomatic disease remain incompletely understood, and the prognostic implications of an asymptomatic presentation are uncertain. Symptom perception may be influenced by multiple demographic, clinical, arrhythmic, structural, functional, and extracardiac factors and may not accurately reflect AF burden or cardiovascular risk. Moreover, the absence of symptoms may delay AF recognition and influence therapeutic management. A systematic synthesis of the available evidence is therefore needed to better define the phenotype of asymptomatic AF, identify factors associated with reduced symptom perception, and clarify its relationship with subsequent clinical outcomes.

Condition being studied The condition of interest is asymptomatic atrial fibrillation (AF), encompassing clinically silent AF as well as subclinical AF identified incidentally or through prolonged electrocardiographic monitoring and cardiac implantable electronic devices. AF may occur without recognizable symptoms despite a substantial arrhythmic burden and underlying cardiovascular disease. The absence of symptoms can delay diagnosis and may result in AF being detected only during routine assessment, continuous rhythm surveillance, or following a cardiovascular complication.

Sonaglioni et al. INPLASY protocol 202690012. doi:10.37766/inplasy2026.9.0012 Downloaded from https://inplasy.com/inplasy-2026-9-0012/

The review investigates the broad clinical phenotype associated with asymptomatic AF, considering demographic, clinical, anthropometric, laboratory, electrocardiographic, structural and functional cardiac, and therapeutic characteristics. Particular attention is given to factors potentially influencing symptom perception, including ventricular response, AF pattern and duration, comorbidity burden, left atrial remodeling and mechanical function, and emerging extracardiac determinants such as thoracic morphology.

The review also evaluates the prognostic significance of an asymptomatic presentation, including its relationship with mortality, thromboembolic events, heart failure, hospitalization, bleeding, AF progression, and other cardiovascular outcomes. Comparisons are primarily made with symptomatic AF, while alternative study-specific comparator populations are considered when relevant to the identification, progression, or prognosis of asymptomatic or subclinical AF.

METHODS

Search strategy A comprehensive electronic literature search was conducted in PubMed/MEDLINE, Scopus, and EMBASE, covering the period from database inception to September 1, 2026. The search was designed to retrieve studies investigating the characteristics and determinants of asymptomatic atrial fibrillation (AF), as well as evidence concerning its clinical course and associated outcomes.

Search terms combined concepts related to AF and symptom status, including “atrial fibrillation”, “asymptomatic atrial fibrillation”, “silent atrial fibrillation”, “subclinical atrial fibrillation”, “symptomatic atrial fibrillation”, “asymptomatic status”, “symptom status”, and “AF symptoms”. These terms were combined with keywords addressing potential associated factors and clinical consequences, including “predictors”, “determinants”, “risk factors”, “clinical characteristics”, “atrial remodeling”, “outcomes”, “prognosis”, “mortality”, “stroke”, “systemic embolism”, “heart failure”, “hospitalization”, “bleeding”, and “atrial fibrillation progression”.

Boolean operators AND and OR were used to combine search concepts, with database-specific adaptations made when necessary. Reference lists of potentially relevant full-text articles and eligible studies were also examined to identify additional publications not retrieved through the electronic searches. No restrictions were imposed according to language, geographical location, or clinical setting.

Participant or population Adult participants (≥18 years) with asymptomatic, silent, or subclinical atrial fibrillation (AF) were considered eligible. The population included individuals with clinically diagnosed AF without AF-related symptoms, patients classified as asymptomatic using symptom-based assessment systems such as the EHRA classification, and individuals in whom subclinical AF was identified through prolonged electrocardiographic monitoring or cardiac implantable electronic devices.

Eligible populations could be drawn from community-based cohorts, hospital settings, AF registries, screening programs, or populations undergoing continuous or device-based rhythm surveillance. Patients with different AF patterns, including first-diagnosed, paroxysmal, persistent, long-standing persistent, or permanent AF, were considered when relevant to the review objectives.

The principal comparison involved patients with symptomatic AF. However, studies including participants without detected AF or other study-specific comparator populations were also eligible when they provided relevant information regarding factors associated with asymptomatic or subclinical AF, its detection or progression, or subsequent clinical outcomes.

Intervention Not applicable. This systematic review does not evaluate a specific therapeutic or diagnostic intervention. Instead, it investigates factors associated with asymptomatic atrial fibrillation (AF) and examines its clinical outcomes. Where reported, pharmacological and non-pharmacological AF management strategies, including anticoagulation, rate- and rhythm-control therapies, electrical cardioversion, and catheter ablation, are considered as clinical or therapeutic characteristics rather than predefined interventions.

Comparator The primary comparator consisted of adult patients with symptomatic atrial fibrillation (AF), allowing direct evaluation of differences in demographic, clinical, anthropometric, laboratory, electrocardiographic, imaging, therapeutic, and prognostic characteristics according to symptom status. Symptomatic AF included patients reporting AF-related manifestations or classified as symptomatic according to the criteria adopted in each individual study.

Sonaglioni et al. INPLASY protocol 202690012. doi:10.37766/inplasy2026.9.0012 Downloaded from https://inplasy.com/inplasy-2026-9-0012/

Studies using alternative comparator groups were also eligible when relevant to the review objectives. These included participants without documented AF or other study-specific reference populations, particularly in studies investigating subclinical or device-detected AF, factors associated with its detection or progression, and subsequent clinical outcomes. Comparator definitions reported by the original studies were retained to account for differences in study populations and AF ascertainment methods.

Study designs to be included Eligible study designs included prospective and retrospective observational cohort studies, multicenter or single-center registries, cross-sectional studies, case-control studies, and relevant post-hoc analyses of clinical trials. Studies were considered eligible when they provided original quantitative data addressing at least one of the predefined review domains: factors associated with asymptomatic or subclinical atrial fibrillation (AF), and/or clinically relevant outcomes associated with an asymptomatic AF presentation.Reviews, systematic reviews, meta-analyses, editorials, commentaries.

Eligibility criteria Studies were eligible when they enrolled adults (≥18 years) and reported original quantitative data addressing at least one of the two main review domains: factors associated with asymptomatic or subclinical atrial fibrillation (AF), and/or clinically relevant outcomes related to an asymptomatic AF presentation. Eligible populations included patients with clinically recognized silent AF, individuals classified as asymptomatic according to symptom-based criteria, and participants with subclinical AF identified through prolonged electrocardiographic or device-based rhythm monitoring.

Studies directly comparing asymptomatic and symptomatic AF were eligible, as were investigations using participants without detected AF or another study-specific reference population when they provided relevant information on the detection, associated factors, progression, or outcomes of asymptomatic/subclinical AF. Eligible studies were required to report extractable information concerning demographic, clinical, anthropometric, laboratory, electrocardiographic, imaging, or therapeutic characteristics and/or relevant clinical endpoints.

Studies were excluded when an asymptomatic or subclinical AF population could not be clearly identified, when quantitative data relevant to the review objectives could not be extracted, or when they represented reviews, meta-analyses, editorials, commentaries, case reports, conference abstracts with insufficient data, or other non-original publications. Reports derived from overlapping cohorts were retained only when they contributed distinct information pertinent to the predefined objectives.

Information sources The principal information sources were PubMed/MEDLINE, Scopus, and EMBASE, which were systematically searched from database inception through September 1, 2026. The electronic search was complemented by manual screening of the reference lists of potentially eligible full-text articles and studies included in the final review to identify additional relevant publications that might not have been retrieved through the database searches. No restrictions were applied according to language, geographical region, or clinical setting.

Main outcome(s) The main outcomes of the review were the identification and characterization of factors associated with asymptomatic atrial fibrillation (AF) and the assessment of clinically relevant outcomes according to AF symptom status. Factors of interest encompassed demographic, clinical, anthropometric, laboratory, electrocardiographic, structural and functional cardiac, and therapeutic characteristics. When available, study-specific adjusted measures of association were preferentially considered.

Clinical outcomes included all-cause and cardiovascular mortality, ischemic stroke, transient ischemic attack, systemic embolism, myocardial infarction or acute coronary syndrome, heart failure and heart failure-related hospitalization, overall and cardiovascular hospitalization, major or clinically relevant bleeding, AF progression or recurrence, and composite cardiovascular or thromboembolic endpoints. Outcomes were evaluated according to the definitions and follow-up periods reported in the individual studies, primarily comparing asymptomatic with symptomatic AF.

Additional outcome(s) Additional outcomes included differences in atrial fibrillation (AF) phenotype and management according to symptom status. These comprised AF pattern and duration, ventricular rate, measures of left atrial structure and mechanical function, circulating biomarkers, thromboembolic and bleeding risk scores, and cardiac device use. Therapeutic outcomes of interest included differences in oral anticoagulation, rate-control and rhythm-control treatment, antiarrhythmic drug use, electrical cardioversion, and catheter ablation.

Sonaglioni et al. INPLASY protocol 202690012. doi:10.37766/inplasy2026.9.0012 Downloaded from https://inplasy.com/inplasy-2026-9-0012/

Where available, the review also considered AF burden and episode characteristics, progression from subclinical or paroxysmal AF toward more sustained forms, and emerging potential determinants of symptom perception, including thoracic morphology. These additional outcomes were analyzed according to their original study definitions and were intended to provide a broader characterization of the asymptomatic AF phenotype and its clinical management.

Data management Data were managed using a predefined standardized spreadsheet specifically developed for this systematic review. For each eligible study, information was systematically recorded on study characteristics, population, sample size, definition and ascertainment of asymptomatic AF, comparator group, follow-up duration, associated factors, therapeutic management, and clinical outcomes. Whenever studies directly compared asymptomatic and symptomatic AF, data were extracted separately for the two groups.

Variables were organized into demographic and anthropometric characteristics, cardiovascular risk factors and comorbidities, AF phenotype, vital parameters, laboratory and biomarker data, electrocardiographic and imaging findings, risk scores, cardiac devices, and pharmacological and non-pharmacological treatments. Study-specific measures of association, including odds ratios and hazard ratios with corresponding confidence intervals or P values, were recorded as originally reported, with preference given to adjusted estimates when available.

Because definitions, measurement methods, populations, and endpoints differed across studies, data were harmonized only when variables were considered clinically comparable. Original study definitions were otherwise preserved. Unreported information was classified as missing and was not imputed.

Quality assessment / Risk of bias analysis Methodological quality and risk of bias were assessed using the National Institutes of Health Quality Assessment Tool for Observational Cohort and Cross-Sectional Studies. The tool examines key domains related to study design and conduct, including clarity of the research question, definition of the study population, participation rate, eligibility criteria, sample-size justification, temporal relationship between exposure and outcome, exposure assessment, repeated exposure measurement, outcome assessment, adequacy and completeness of follow-up, and adjustment for potential confounding.

Each applicable domain was rated as Yes, No, Not Reported, or Not Applicable according to the information provided in the original publication. The number of criteria fulfilled was considered in relation to the total number of applicable items, and an overall methodological quality judgment of Good, Fair, or Poor was assigned to each study. The final rating was based on the overall pattern of methodological strengths and limitations rather than on any single criterion.

Strategy of data synthesis Given the substantial clinical and methodological heterogeneity across the eligible studies, a formal meta-analysis was not planned. Evidence was instead synthesized using structured descriptive and exploratory quantitative approaches. For each variable, the number of contributing studies and the corresponding number of participants with available data were recorded separately for asymptomatic and symptomatic AF.

For continuous variables, study-level estimates were combined using sample-size weighting, and the resulting weighted values were accompanied by the range of central estimates across contributing studies. When means, standard deviations, and sample sizes were available, these data were used for exploratory comparisons between groups. If variability was reported as an interquartile range rather than a standard deviation, appropriate statistical approximations were used when feasible. Categorical variables were summarized by aggregating reported events and denominators and expressing them as proportions.

Study-specific associations with asymptomatic AF and clinical outcomes were retained in their originally reported metrics, including odds ratios, adjusted odds ratios, hazard ratios, and adjusted hazard ratios with corresponding 95% confidence intervals. These estimates were displayed graphically to facilitate comparison of their direction and magnitude, without calculating pooled summary effects across heterogeneous populations, comparators, effect measures, or follow-up periods. Accordingly, all between-group statistical comparisons were considered exploratory and hypothesis-generating rather than confirmatory.

Subgroup analysis No formal subgroup meta-analysis was planned because of the substantial heterogeneity in study populations, definitions of asymptomatic AF, comparator groups, effect

Sonaglioni et al. INPLASY protocol 202690012. doi:10.37766/inplasy2026.9.0012 Downloaded from https://inplasy.com/inplasy-2026-9-0012/

measures, and follow-up durations. Where sufficient information was available, findings were examined descriptively according to clinically relevant study characteristics and AF phenotypes.

Exploratory subgroup assessment included differences according to AF pattern, particularly paroxysmal versus persistent, long-standing persistent, or permanent AF, as well as clinically diagnosed versus subclinical or device-detected AF. Findings were also considered according to the clinical setting and method of AF detection when these characteristics could influence symptom recognition or prognosis. Study-specific subgroup estimates were retained in the form reported by the original investigators and were not statistically pooled across heterogeneous subgroups. These analyses were intended to explore potential sources of variability in the factors associated with asymptomatic AF and its clinical outcomes rather than to provide confirmatory subgroup comparisons.

Sensitivity analysis No formal sensitivity analysis was planned because the substantial clinical and methodological heterogeneity of the included studies precluded calculation of pooled summary estimates. Accordingly, there was no overall meta-analytic effect estimate on which conventional leave-one-out or study-exclusion sensitivity analyses could be performed.

The robustness of the findings was instead considered qualitatively by examining the consistency and direction of study-specific associations across different populations, AF definitions, comparator groups, study designs, and follow-up periods. Particular caution was applied to findings supported by only one or a small number of studies and to results derived from highly selected populations. Adjusted effect estimates were preferentially considered when available, while unadjusted findings were interpreted separately. Therefore, the synthesis was primarily descriptive and exploratory, and potentially heterogeneous or isolated findings were interpreted as hypothesis-generating rather than definitive.

Language restriction No language restrictions were applied. Studies were considered eligible irrespective of the language of publication.

Country(ies) involved Italy.

Other relevant information The review was conducted and reported in accordance with the PRISMA 2020 framework. Because of substantial heterogeneity in study populations, definitions of asymptomatic and subclinical AF, methods of arrhythmia detection, comparator groups, outcome definitions, and follow-up duration, the evidence was synthesized primarily through structured qualitative and exploratory quantitative analyses rather than formal meta-analysis.

Particular attention was given to preserving the clinical context of individual studies and distinguishing descriptive findings from adjusted associations. Emerging and less extensively investigated aspects of the asymptomatic AF phenotype, including left atrial mechanical function, circulating biomarkers, AF episode characteristics, and thoracic morphology, were also considered when relevant data were available. These findings were interpreted as exploratory when supported by limited evidence.

Keywords atrial fibrillation; asymptomatic atrial fibrillation; silent atrial fibrillation; subclinical atrial fibrillation; symptom status; atrial remodeling; cardiovascular outcomes; heart failure; thoracic.

Dissemination plans The findings of this systematic review are planned to be disseminated through publication in a peer-reviewed scientific journal. The results may also be presented at national or international scientific meetings and congresses in the fields of cardiology, cardiac electrophysiology, and cardiovascular imaging. The aim of dissemination will be to increase awareness of the clinical phenotype, associated factors, and prognostic implications of asymptomatic atrial fibrillation and to highlight areas requiring further prospective investigation.

Contributions of each author
Author 1 - Andrea Sonaglioni - Drafted the manuscript.
Email: sonaglioniandrea@gmail.com
Author 2 - Giulio Francesco Gramaglia- The author contributed to the development of the search strategy.
Email: giulio.gramaglia@unimi.it
Author 3 - Elena Mondazzi- The author contributed to the development of the search strategy.
Email: elena.mondazzi@unimi.it
Author 4 - Gian Luigi Nicolosi- The author read, provided feedback and approved the final manuscript.
Email: gianluigi.nicolosi@gmail.com
Author 5 - Michele Lombardo- The author read, provided feedback and approved the final manuscript.
Email: michele.lombardo@multimedica.it

Sonaglioni et al. INPLASY protocol 202690012. doi:10.37766/inplasy2026.9.0012 Downloaded from https://inplasy.com/inplasy-2026-9-0012/

Author 6 - Massimo Baravelli- The author read, provided feedback and approved the final manuscript.
Email: massimo.baravelli@multimedica.it

Sonaglioni et al. INPLASY protocol 202690012. doi:10.37766/inplasy2026.9.0012 Downloaded from https://inplasy.com/inplasy-2026-9-0012/
